# 二代测序技术在NSCLC中的临床应用中国专家共识(2020版)

**DOI:** 10.3779/j.issn.1009-3419.2020.101.45

**Published:** 2020-09-20

**Authors:** 中国临床肿瘤学会非小细胞肺癌专家委员会

## 1 引言

肺癌是目前全球最常见、致死率最高的恶性肿瘤^[1]^。2018年全球肺癌新发病例近209.4万例，占所有恶性肿瘤的11.6%，死亡病例176.1万例，占所有恶性肿瘤的18.4%^[1]^。全国肿瘤登记中心数据^[2]^显示，2014年我国新发肺癌患者数为78.1万，死亡数达到62.6万，居所有恶性肿瘤发病和死亡人数首位。17个癌症注册中心的数据^[3]^分析显示，我国肺癌患者的年龄标化5年相对生存率由2003年-2005年的16.1%仅增加至2012年-2015年的19.7%，仍不乐观。非小细胞肺癌(non-small cell lung cancer, NSCLC)是肺癌中最常见的组织学类型，在肺癌病例中占比超过80%^[4]^。

随着对疾病认识的不断加深，肿瘤的治疗模式也在发生变革，个体化精准治疗模式逐渐成为主流。在NSCLC领域，随着基因检测技术的进步和一系列新药临床研究的突破，近10年间新发现的肿瘤驱动基因不断增多，推动了NSCLC靶向治疗药物的研发和临床应用。

近年来兴起的免疫治疗是肿瘤治疗领域的革命性突破。NSCLC的免疫治疗研发和应用速度进步显著，目前已有多个针对NSCLC患者的免疫治疗方案获批。但如何筛选出可从免疫治疗中获益的人群，仍是该疗法在临床应用中的一大挑战。研究^[5]^表明，全面的分子生物学检测信息可为肺癌患者免疫治疗的方案选择、预后判断，以及为临床试验入组提供依据。

在此背景下，传统基因检测方法因基因覆盖有限，存在漏检的可能性，以及因组织样本耗竭而影响后续检测等方面的缺陷，已不能充分满足目前的临床需求。因此全面的肿瘤相关基因检测方法则成为提升NSCLC患者治疗效果及预后的有效手段。中国临床肿瘤学会(Chinese Society of Clinical Oncology, CSCO)NSCLC诊疗指南也提出应采用经过验证的检测方法同时检测多个驱动基因^[6]^。美国国家综合癌症网络(National Comprehensive Cancer Network, NCCN)NSCLC指南则强烈支持通过全面分子测序的结果，指导临床治疗方案的确定，或纳入相关临床试验中。其他多个肺癌诊疗指南^[6-8]^也提出了类似的建议。

二代基因测序(next generation sequencing, NGS)又称为高通量测序，该技术能够同时对上百万甚至数十亿个DNA进行分析，实现了高通量测序的目标。目前国外已发表了多个NGS检测技术指南，其中颇具影响力的包括EuroGentest和欧盟人类遗传学会在2015年联合发布的《二代测序诊断指南》^[9]^；美国分子病理学会(Association for Molecular Pathology, AMP)和美国病理学家协会(College of American Pathologists, CAP)在2017年联合发布的《基于二代测序的肿瘤panels验证指南》^[10]^。我国近年来也发表了多个NGS专家共识，包括2017年中华医学会病理学分会和中国抗癌协会肿瘤病理专业委员会联合发布的《临床分子病理实验室二代基因测序检测专家共识》^[11]^；2018年陆续发表了由国内多名专家和机构联合撰写的《临床基因检测报告规范与基因检测行业共识探讨》^[12]^、中华医学会制订的《二代测序技术在肿瘤精准医学诊断中的应用专家共识》^[13]^，以及中国抗癌协会血液肿瘤专业委员会、中华医学会血液学分会、中华医学会病理学分会联合制订的《二代测序技术在血液肿瘤中的应用中国专家共识(2018年版)》^[14]^等。

现有的这些NGS指南通常侧重检验科室的技术参数标准，尚缺乏可指导NGS检测在实体瘤临床诊疗路径中应用的指南或共识，然而我国NSCLC患者数量庞大，精准治疗药物的可及性高，全面而准确的肿瘤基因诊断结果已经成为肺癌医师临床诊疗的刚需。因此，我们邀请行业内资深专家共同讨论并撰写了本共识，旨在为我国NSCLC临床诊疗规范使用NGS这一新型基因检测技术提供指引。

## 2 NGS检测的适用人群

### 2.1 常规推荐进行NGS检测的NSCLC患者

当患者确诊为晚期或转移性NSCLC后，就需要考虑进行分子病理检测，以指导后续治疗和判断预后。基于临床研究中报道的分子靶向药物和免疫药物治疗效果，CSCO NSCLC诊疗指南(2020)推荐对病理学诊断为非鳞癌的肺癌患者组织标本进行分子标志物检测^[6]^。

针对肺鳞癌患者，目前尚无明确的基因检测位点和获批的分子靶向药物，也无临床证据支持对单纯肺鳞癌患者常规进行某个基因变异的检测。虽然单纯肺鳞癌患者中有4%的表皮生长因子受体(epidermal growth factor receptor, *EGFR*)突变率^[15]^，但尚无证据支持肺鳞癌患者使用靶向EGFR酪氨酸激酶抑制剂(tyrosine kinase inhibitors, TKI)显著获益，因此不推荐对单纯肺鳞癌患者进行*EGFR*基因检测。而另一方面，研究^[16, 17]^提示病理类型是含有腺癌成分或具有腺癌分化的混合鳞癌的NSCLC患者也可能携带敏感型突变，可从靶向治疗中获益。一些临床特征也与敏感型突变相关，如较年轻、不吸烟或少吸烟(少于100支/年)^[7]^。因此CSCO NSCLC诊疗指南(2020)也建议对不吸烟、经小标本活检诊断为肺鳞癌或混合腺癌成分的患者进行驱动基因检测^[6]^。

**共识1：推荐所有病理诊断为肺腺癌、含有腺癌成分的肺癌以及不能分型的晚期新发或术后复发的NSCLC患者常规进行基因检测。【I级推荐】**

**对经小标本活检诊断为含有腺癌成分或具有腺癌分化的混合型鳞癌，以及年轻或不吸烟/少吸烟肺鳞癌患者，也推荐进行基因检测。【II级推荐】**

### 2.2 NGS和传统检测方法的比较

随着对NSCLC发生发展机制认识的逐渐深入，在研的新靶点和治疗方案也不断增多，加上临床诊疗需求的不断拓展，对NSCLC的肿瘤基因检测技术提出了更高的要求^[13]^。

目前，在我国NSCLC的临床诊疗过程中，筛选分子标志物所使用的检测方法仍以免疫组织化学检测(immunohistochemistry, IHC)、荧光原位杂交检测(fluorescence *in situ* hybridization, FISH)和聚合酶链反应(polymerase chain reaction, PCR)等传统基因检测技术为主。

IHC是利用抗原抗体反应和化学显色原理，检测组织切片或细胞标本中特定蛋白质表达量的技术，与其他技术检测DNA变异有所不同。IHC技术成熟，成本低廉，但仅能检测蛋白质表达量的改变，并且由于其依赖于人工判读，对于1+/2+的弱阳性结果不能直接确诊，需要使用其他方法再次检测进行验证。

FISH是通过荧光标记的DNA探针与细胞核内的DNA靶序列杂交，并在荧光显微镜下观察并分析基因扩增或融合的一种分子遗传学分析技术。FISH是临床检测基因扩增和融合的金标准，但由于其操作复杂，依赖人工判读，因此对诊断医生要求较高。同时，由于FISH需要利用预先设定好的探针进行杂交，仅能检测少数已知的融合基因，无法确定和区分不同的融合配体基因，更无法发现新伴侣的融合基因。

PCR和NGS都是检测DNA变异的技术。临床上最为广泛应用的是扩增阻滞突变系统(amplification refractory mutation system, ARMS)-PCR技术。ARMS-PCR检测具有单次检测DNA使用量少，灵敏度高(0.1%-0.001%)，操作简单，检测周期短(几小时内可出具检测报告)，单个基因/位点的检测费用较低，可以同时检测数十个已知的基因点突变、小片段插入/缺失、部分明确的融合基因等特点。与ARMS-PCR相比，NGS检测存在的缺点包括：所需DNA样本量较高，操作流程复杂(包含杂交捕获、建库、测序、数据分析、变异注释等流程)，对于操作人员及操作环境的要求高，检测周期长(针对涵盖基因数量不同的检测panel，需3 d-10 d出报告)，费用相对较高等；但NGS检测技术具有其不可替代的优势：可以同时涵盖数十至数百个基因，检测所有位点突变、未知伴侣的融合基因、拷贝数变异等多种变异类型^[18]^，同时可评估肿瘤突变负荷(tumor mutation burden, TMB)和微卫星不稳定性(microsatellite instability, MSI)等免疫治疗相关分子标志物。

传统基因检测方法因临床可及性高和单次检测成本较低而在临床应用广泛。但与NGS相比，传统检测技术也存在一些技术缺陷，主要体现在：①通量低，一次只能筛查出有限数量的基因变异，会遗漏某些类型的基因变异；②不能检测未知基因变异；③不能同时提供TMB和MSI数据；④耗竭样本，影响后续进一步检测。尤为重要的是，这类检测方法可能漏检少见或罕见的突变，导致“假阴性”结果，使患者失去精准治疗的机会。例如，PCR仅限于分析DNA的点突变、小片段插入/缺失、少数常见亚型的融合基因检测^[23]^，ARMS-PCR无法有效检出*EGFR* 19del某些罕见亚型^[19]^；ARMS-PCR同样无法有效检出酪氨酸蛋白激酶*MET*(c-mesenchymal-epithelial transition factor, *C-MET*)14号外显子跳跃突变等罕见基因变异^[20]^；IHC可检测蛋白表达，但无法检测任何已知或未知的突变^[21]^；FISH可检测拷贝数变异和重排(融合)，但无法检测碱基替换或插入/缺失^[22]^。

相比传统基因检测方法，NGS检测有明显的技术优势。NGS可以一次性、特定时间(约10个工作日)产生覆盖基因组特定区域(从数个基因到数百个基因以至全外显子组或全基因组)的高通量测序数据^[13]^，在临床诊疗中有重要的应用价值：①NGS检测可同时检出多个基因的点突变、插入/缺失、拷贝数变异、基因重排四种变异形式，提供全面的基因突变数据，为初诊晚期NSCLC患者提供精准用药指导，包括正确选择靶向治疗方案、协助判断是否可使用免疫治疗方案、指导临床试验入组、提供疗效预测信息等^[24]^；②避免由于单个基因序贯检测带来的样本耗竭和检测时间延长，更加快速地为患者治疗方案选择提供依据；③当患者出现疾病进展时再次进行全面的基因检测，有助于发现潜在的耐药机制，并且全面的基因变异检测信息可以为下一步治疗方案的选择提供依据^[24]^；④尽管NGS多基因同时分析的总成本较高，但对比传统检测方法，单个基因单个位点的平均检测费用已大大下降。

由于NGS技术可以同时检测多个基因所有变异类型的特点，因此对于传统检测方式阴性的患者样本建议使用NGS复检。一项针对晚期NSCLC患者的回顾性研究^[25]^数据显示，83%的患者携带潜在可用药的肿瘤驱动基因突变，其中50%为指南推荐的肺癌可用药基因变异；在既往传统单基因检测提示*EGFR*和间变性淋巴瘤激酶(anaplastic lymphoma kinase, *ALK*)变异阴性的晚期NSCLC患者中，经NGS检测发现17.4%的患者携带至少一个*EGFR*或*ALK*基因变异。另一项研究^[26]^发现，基于NGS检测筛选出的47例*ALK*重排阳性肿瘤样本(41例*EML4-ALK*融合，6例其他融合伴侣)，对有既往FISH检测结果的31例肿瘤样本进行分析，显示有11例(35%)患者在既往FISH检测中为假阴性。这11例患者中有9例基于NGS检测结果接受了克唑替尼治疗并实现临床获益。纪念斯隆-凯特琳癌症中心的研究^[27]^中，研究者对非NGS技术检测显示基因变异阴性的31例肺腺癌患者的组织样本使用FoundationOne^®^CDx重新进行NGS分析，结果有65%的样本检出存在可用药的基因变异，部分患者基于检测结果接受了已获批或NCCN指南推荐的靶向药物治疗，并达到临床获益。一项韩国的研究^[28]^对51例采用非NGS检测提示*EGFR/KRAS*(kirsten rat sarcoma viral oncogene)*/ALK*阴性的肺腺癌样本分析后发现，31%的患者存在NCCN指南推荐的可用药靶点基因变异。由此可见，非NGS技术检测驱动基因变异存在“假阴性”的可能性，因此对于传统检测方式阴性的患者样本建议使用NGS复检。

**共识2：针对敏感型突变发生率高的NSCLC患者(见“共识1”)，常规基因检测结果为阴性时，建议使用中国国家药品监督管理局(National Medical Products Administration, NMPA)或美国食品药品监督管理局(Food and Drug Administration, FDA)批准的NGS产品进行复检。【III级推荐】**

## 3 晚期新发或术后复发NSCLC患者首次进行基因检测的共识意见

在过去20余年间，晚期NSCLC的临床治疗从只有一种针对*EGFR*突变的靶向治疗方案，发展至今已有针对不同驱动基因变异的多种分子靶向治疗方案。分子靶向治疗已成为驱动基因变异阳性肺癌患者的标准治疗策略之一。另一方面，以程序性死亡受体1(programmed death-1, PD-1)/PD配体1(PD ligand 1, PD-L1)抑制剂等免疫检查点抑制剂为代表的新型肿瘤免疫疗法近年来在晚期恶性肿瘤治疗领域发展迅速，已成为部分驱动基因阴性的晚期NSCLC患者的重要治疗方案之一。与此同时，基于分子生物学探索分子靶向治疗和免疫治疗生物标志物，筛选治疗优势人群成为研究热点。国内外的肺癌诊疗指南^[5, 6]^也一致推荐对NSCLC患者进行广泛的分子标记物检测，并制定了基于检测结果的个体化精准诊疗流程。

### 3.1 晚期新发或术后复发NSCLC患者首次进行基因检测的目标范围推荐

随着越来越多针对NSCLC患者的分子靶向和免疫治疗方案的获批，国内外NSCLC诊疗指南推荐检测的NSCLC驱动基因和生物标志物也不断增加。国内外NSCLC诊疗指南^[5, 6]^对不可手术的III期及IV期NSCLC患者推荐的分子标志物检测内容和相关个体化治疗策略见表 1-表 3。

**1:** 国内外肺癌诊疗指南对不可手术Ⅲ期及Ⅳ期患者推荐的首次分子标志物检测内容 First-time molecular biomarker testing for advanced NSCLC patients recommended by CSCO and NCCN NSCLC guidelines

	常规检测的基因变异	具有临床意义的基因变异	免疫治疗生物标志物
CSCO NSCLC诊疗指南(2020)^[6]^	− *EGFR*突变 − *ALK*融合 − *ROS1*融合 (非鳞癌NSCLC I级推荐；不吸烟、经小标本活检诊断鳞癌或混合腺癌II级推荐)	− *KRAS*突变 − *ERBB2*(*HER2*)扩增/突变 − *BRAF* V600E突变 − *RET*重排 − *MET*扩增和*MET* 14号外显子跳跃突变 − *NTRK*融合 (II级推荐)	− PD-L1表达水平(I级推荐) − TMB(III级推荐)
NCCN NSCLC诊疗指南 (V6. 2020^[5]^)	− *EGFR*突变(非鳞癌NSCLC 1类推荐) − *ALK*融合(非鳞癌NSCLC 1类推荐) − *ROS1*融合 − *BRAF*突变 − *MET* 14号外显子跳跃突变 − *RET*重排	− *NTRK*融合 − *MET*扩增 − *ERBB2*(*HER2*)突变	− PD-L1表达水平(1类推荐) − TMB
NCCN指南推荐证据等级，无特殊指明均为2A类推荐。NCCN：国家综合癌症网络；EGFR：表皮生长因子受体；NSCLC：非小细胞肺癌；CSCO：中国临床肿瘤学会；ALK：间变性淋巴瘤激酶；PD-1：程序性死亡受体1；PD-L1：PD配体1；TMB：肿瘤突变负荷。			

**2:** 2020版CSCO NSCLC诊疗指南(2020)对分子标志物检测结果阳性的不可手术Ⅲ期及Ⅳ期患者推荐的一线分子靶向/免疫治疗方案^[6]^ First-line therapy for molecular biomarker positive advanced NSCLC patients recommended by CSCO NSCLC guideline^[6]^

生物标志物	一线治疗方案
*EGFR*敏感突变阳性	− I级推荐：阿法替尼、厄洛替尼、达克替尼、吉非替尼、奥希替尼、埃克替尼(1A类证据) − II级推荐：吉非替尼/厄洛替尼+化疗(PS 0-1)；厄洛替尼+贝伐珠单抗
*ALK*融合阳性	− I级推荐：阿来替尼(优先推荐，1A类证据)；克唑替尼(1A类证据) − III级推荐：Brigatinib(1A类证据)
*ROS1*重排阳性	− I级推荐：克唑替尼(1类证据) − III级推荐：恩曲替尼(Entrectinib)
*BRAF* V600E突变阳性	− III级推荐：达拉非尼+曲美替尼/达拉非尼(3类证据)
*NTRK*基因融合阳性	− III级推荐：拉罗替尼(Larotrectinib)或恩曲替尼(Entrectinib)(3类证据)
PD-L1表达阳性	IV期无驱动基因、非鳞癌NSCLC治疗 − I级推荐：帕博利珠单抗单药[限PD-L1 TPS≥50%(1A类证据)，PD-L1 TPS 1%-49%(2A类证据)]；帕博利珠单抗联合培美曲塞和铂类(1A类证据) − II级推荐：卡瑞利珠单抗联合培美曲塞和铂类(1A类证据)；紫杉醇+卡铂+贝伐珠单抗+阿替利珠单抗(1A类证据)；白蛋白紫杉醇+卡铂+阿替利珠单抗(1A类证据) 无驱动基因、IV期鳞癌的治疗 − I级推荐：帕博利珠单抗单药[PD-L1 TPS≥50%(1A类证据)，PD-L1 TPS 1%-49%(2A类证据)]；帕博利珠单抗联合紫杉醇/白蛋白紫杉醇和铂类(1A类证据)
PS：体能状态；TPS：肿瘤比例评分。	

**3:** NCCN指南中对于其他相对少见基因变异型NSCLC患者推荐的靶向/免疫治疗方案(在我国相关药物适应证尚未获批)^[5]^ Appropriate treatment options for other molecular biomarker positive patients recommended by NCCN NSCLC guidelines^[5]^

生物标志物	推荐靶向/免疫治疗方案
*MET*高水平扩增	-克唑替尼
*MET* 14号外显子跳跃突变	- Capmatinib、克唑替尼
*RET*重排	- Selpercatinib、卡博替尼、Vandetanib
*ERBB2*(*HER2*)突变	-曲妥珠单抗-美坦新偶联物(Ado-trastuzumab emtansine)
高TMB	-纳武利尤单抗单药或联合Ipilimumab
NCCN指南推荐证据等级，无特殊指明均为2A类推荐。	

### 3.2 NSCLC常见驱动基因

考虑到中国NSCLC患者特有的基因变异频率、药物在中国的获批适应证及药物可及性，CSCO NSCLC诊疗指南(2020)明确指出对不可手术的晚期NSCLC患者推荐的首次分子标志物检测内容必须包含*EGFR*、*ALK*、*ROS1*(ROS proto-oncogene 1)三个基因(表 1)。*EGFR*、*ALK*、*ROS1*常见变异涵盖多个位点及多种变异类型，本共识推荐使用NMPA批准的检测产品，无论使用PCR、FISH或NGS方法，建议同时检测*EGFR*突变、*ALK*融合和*ROS*1融合三种形式的基因变异。

*EGFR*：*EGFR*突变是NSCLC驱动基因研究中最早发现的基因突变，也是NSCLC中最常见的驱动基因变异。东亚患者中*EGFR*突变的发生率高达46.7%，尤其见于腺癌(54.1%)和女性(61.8%)患者^[29]^。该基因的常见突变位点发生在18号-21号外显子上，其中最常见的是19号外显子缺失突变(19del)以及21号外显子L858点突变(L858R)，这两种突变占比约为90%，均为EGFR-TKI敏感型突变^[5]^。分子生物学研究揭示了一些*EGFR*少见突变(发生率约10%)与EGFR-TKI治疗敏感相关，如19号外显子插入、L861Q、G719X、S768I、20号外显子插入突变A763_Y764insFQEA；研究^[5]^还发现了少见的耐药突变，如20号外显子其他插入类型(除A763_Y764insFQEA)和T790M(对三代TKI敏感)。一项基于FoundationOne^®^CDx检测结果^[30]^的数据分析，描述了NSCLC中罕见*EGFR*突变亚型的分布特征，发现在非L858R/del19的*EGFR*突变中，20号外显子插入突变和G719X突变最为常见。

*ALK*：*ALK*基因融合在NSCLC中的发生率约为7%，*EML4*是最常见的*ALK*融合伴侣，占*ALK*重排的90%-95%^[31]^。*EML4-ALK*又分为多个亚型，其中V1型(E13; A20)和V3型(E6; A20)占比最高，均为32%左右^[31]^，其他*EML4-ALK*融合亚型则较为少见(占比均不足10%)^[31]^。除*EML4*这一最常见的融合伴侣外，研究^[32]^还发现*KIF5B*、*TFG*、*KLC1*、*SOCS5*、*HIP1*、*TPR*、*BIRC6*等多种罕见的*ALK*融合伴侣，这些基因变异可能与肿瘤进展和治疗相关。不同融合亚型或不同治疗方案^[33]^治疗后产生的耐药突变也存在差异^[31]^。

*ROS1*：*ROS1*融合基因在NSCLC中的发生率约为2%^[5]^，重排位点主要发生在32号-36号外显子^[34]^，最常见的*ROS1*融合伴侣是*CD74*、*SLC34A2*、*CCDC6*和*FIG*^[5]^。近年来发现了更多的新型*ROS1*融合伴侣，如基于FoundationOne^®^CDx检出的罕见的新型*TPD52L1-ROS1*融合^[35]^和*TMEM106B-ROS1*融合^[36]^。

**共识3：针对晚期新发或术后复发的NSCLC患者，首次检测建议采用NMPA批准的检测产品，检测至少包括NSCLC常见驱动基因: *EGFR*突变(应涵盖18号、19号、20号、21号外显子)，以及*ALK*融合、*ROS1*融合。【I级推荐】**

### 3.3 NSCLC少见驱动基因

除了上述常见*EGFR*突变、*ALK*融合、*ROS1*融合外，虽然*BRAF*(B-Raf proto-oncogene)、*KRAS*、神经营养因子受体酪氨酸激酶(neurotrophic tyrosine receptor kinase, *NTRK*)*1/2/3*、*MET*、*ERBB2*(receptor tyrosine-protein kinase erbB-2)和*RET*(ret proto-oncogene)等基因在NSCLC中的变异频率相对较低，但由于其已有对应获批或在研的靶向治疗药物，成为NSCLC患者潜在的可靶向治疗基因。虽然目前国内相关靶向药物的可及性较低，但具有前瞻性临床应用价值，应重视对这些靶点的检出。2020版CSCO NSCLC诊疗指南也推荐在广泛分子标志物检测中纳入这些少见驱动基因^[6]^。考虑到上述少见肺癌驱动基因并非全部含有NMPA批准的传统检测产品(IHC、FISH、PCR等)，并且NMPA批准的NGS检测产品(国家食品药品监督管理局网站https://www.nmpa.gov.cn/)也无法涵盖上述全部基因的变异类型，本共识建议结合患者实际临床情况，优先使用NMPA批准的传统或NGS检测产品，在NMPA批准的检测产品无法满足临床需求的情况下可以考虑使用FDA批准的NGS检测产品进行检测，建议检测包括*BRAF* V600E、*KRAS*(如G12C等)、*NTRK1/2/3*融合、*MET* 14号外显子跳跃突变和*MET*扩增、*ERBB2* 20号外显子插入、*RET*融合等少见驱动基因。

*BRAF*：2%-4%的NSCLC患者携带*BRAF*突变^[37]^。*BRAF*突变分为3种类型，I类(*BRAF* V600E/K/D/R/M)突变是最常见于NSCLC的*BRAF*突变，NCCN指南^[5]^推荐的靶向药物为达拉菲尼和曲美替尼。II类、III类*BRAF*突变患者脑转移发生率高，共突变发生率高，暂无确定的靶向治疗方案，并且患者预后通常较差^[37]^。

*KRAS*：*KRAS*是NSCLC的一个重要驱动基因，10.1%的东亚NSCLC患者携带这一突变^[29]^。*KRAS*突变型NSCLC患者EGFR-TKI疗效不佳，预后较差^[5]^。由于*KRAS*突变类型多，下游信号通路复杂，既往的靶向药物研究均宣告失败，目前并无推荐的靶向药物^[6]^。*KRAS* G12C是一个潜在可用药靶点。NSCLC患者中约20%的*KRAS*突变为*KRAS* G12C突变。目前针对这一突变类型的靶向药物虽然尚未获批，但两个*KRAS* G12C抑制剂——AMG510^[38]^和MRTX849^[39]^已在临床前和初期临床试验中显示出良好的治疗效果。从2019年ASCO大会上发布的AMG510 I期临床研究数据来看，这一*KRAS* G12C不可逆抑制剂在NSCLC患者中的疗效和安全性均表现良好^[40]^。

*NTRK*：*NTRK*基因包括*NTRK1/2/3*三种亚型，分别编码TRK A/B/C蛋白。*NTRK*基因发生融合激活后，将引发信号级联反应，驱动促进肿瘤的发生发展。虽然NTRK融合在肺癌患者中的发生率较低(< 1%-5%)^[41]^，但基于NGS检出的NTRK融合肺癌患者在两个泛TRK抑制剂——恩曲替尼(Enctrectinib)^[42]^和拉罗替尼(Larotrectinib)^[43]^的关键篮子研究(basket trial)中均获得显著临床获益。基于这两项研究结果，CSCO NSCLC诊疗指南(2020)将这两个*NTRK*抑制剂推荐用于治疗*NTRK*基因融合阳性的NSCLC患者^[6]^。

*MET*：*c-MET*是NSCLC的重要驱动基因，c-MET通路异常激活主要包括*MET* 14号外显子跳跃突变、*MET*扩增和MET蛋白过表达3种类型。*MET* 14号外显子跳跃突变在肺腺癌中的发生率约为3%，是重要的原发致癌驱动基因变异^[44]^。*MET*扩增在NSCLC中的发生率为3%-7%^[45]^，是三代EGFR-TKI的重要耐药机制之一^[46]^。基于临床研究^[47, 48]^，NCCN指南推荐克唑替尼用于治疗*MET*高水平扩增或*MET* 14号外显子跳跃突变的NSCLC患者^[5]^。此外，已有多个靶向治疗方案在临床研究中显示对*MET*扩增NSCLC有治疗活性^[49-52]^。针对*MET* 14号外显子跳跃突变晚期NSCLC患者的多个新药也已处于临床研究阶段^[53-55]^。

*ERBB2*(*HER2*)：*HER2*突变在NSCLC中以酪氨酸激酶域的18号-21号外显子突变较多，发生率约为2%-4%^[56-58]^。在临床研究中显示对*HER2*突变型NSCLC有治疗活性的药物包括曲妥珠单抗-美坦新偶联物(Trastuzumab Emtansine, T-DM1)^[59]^、Trastuzumab Deruxtecan(T-DXd、DS-8021)^[60]^、吡咯替尼^[61]^、波奇替尼^[62]^、曲妥珠单抗^[63]^、阿法替尼^[64]^和Neratinib^[65]^等。曲妥珠单抗和阿法替尼单药方案既往曾被推荐用于*HER2*突变阳性的NSCLC患者，但因为治疗有效率低，证据级别较低而不再推荐。基于一项II期篮子研究结果^[59]^，NCCN指南目前推荐T-DM1用于治疗*HER2*突变阳性的NSCLC患者^[5]^。

*RET*：*RET*融合在NSCLC中的发生率约为2%^[66]^，变异类型主要包括与*KIF5B*、*TRIM33*、*CCDC6*和*NCOA4*等基因发生融合，以及M918T等位点的点突变。NCCN指南目前推荐多靶点酪氨酸激酶抑制剂卡博替尼和Vandetanib用于治疗*RET*融合突变阳性NSCLC患者^[5]^。但从目前的数据来看，卡博替尼^[67, 68]^和Vandetanib^[69]^对*RET*靶点的治疗效果并不理想。正在开展的临床研究中，阿来替尼^[70]^、多靶点酪氨酸激酶抑制剂[仑伐替尼^[71]^、RXDX-105^[72]^、Pralsenitinib(BLU-667)^[73]^]、RET单靶点抑制剂Selpercatinib(LOXO-292)^[74]^的临床应用数据值得期待。

**共识4：针对晚期新发或术后复发的NSCLC患者，结合患者实际临床情况，如需获得更多的潜在靶点信息，首次检测建议采用NMPA或FDA批准的检测产品，检测包括*BRAF* V600E、KRAS(如G12C等)、*NTRK1/2/3*融合、*MET* 14号外显子跳跃突变和*MET*扩增、*ERBB2* 20号外显子插入、*RET*融合等少见驱动基因变异。【II级推荐】**

### 3.4 NSCLC常见/少见驱动基因的罕见变异形式

随着NGS检测技术在NSCLC临床诊疗中应用的普及，也推动了NSCLC驱动基因研究的迅猛发展，越来越多常见/少见驱动基因的罕见变异形式被揭示与患者的临床疗效密切相关。肿瘤分子标志物研究的目的是为了使越来越多的患者获益于个体化治疗。因此，即使携带罕见基因变异类型的NSCLC患者人群较少，且多为个案报告，但仍需重视其检测，以增加更多患者获得精准治疗的机会，同时也为肺癌新药的研发提供证据支持。

*EGFR*激酶区串联重复(kinase domain duplication, KDD)和融合：*EGFR* KDD在肺癌中发生率约为0.07%，对阿法替尼、埃克替尼等EGFR-TKI治疗敏感^[75, 76]^；*EGFR*融合在肺癌中的发生率为0.08%，一系列*EGFR*融合变异(已报告的有*EGFR-RAD51*、*EGFR-PURB*、*EGFR-SEPT14*、*EGFR-TNS3*、*EGFR-ZCCHC6*、*LINCO1446-EGFR*)可以从厄洛替尼、阿法替尼、埃克替尼治疗中获益^[77]^。

*BRAF* KDD和融合：NSCLC中*BRAF* KDD和*BRAF*融合的发生率不到0.5%^[78, 79]^。个案报告^[79]^显示，携带*BRAF* KDD的NSCLC患者接受BRAF-TKI治疗有效。其他癌种的个案报告提示，针对不同*BRAF*融合基因使用个体化靶向治疗方案可使患者获益^[80, 81]^。

*MET* KDD和融合：*MET* KDD和融合在中国NSCLC患者中的发生率分别为0.09%^[79]^和0.04%^[82]^。携带*MET* KDD的NSCLC患者对MET-TKI治疗有反应^[80]^。国内外多个个案报告^[76, 82-84]^显示，携带*MET*融合的NSCLC患者使用克唑替尼治疗有效。

*ERBB2*(*HER2*)融合：分析数据显示，中国NSCLC患者中*ERBB2*融合的发生率为0.29%。部分患者使用阿法替尼治疗有效^[85]^。

**共识5：共识3和共识4中基因检测均为阴性的NSCLC患者再次检测时，建议采用NMPA或FDA批准的NGS产品，检测包含*EGFR*罕见变异形式(包括激酶区重复和融合)、*BRAF*罕见变异形式(包括激酶区重复和融合)、*MET*罕见变异形式(包括激酶区重复和融合)、*ERBB2*融合等罕见变异形式。【III级推荐】**

### 3.5 免疫治疗疗效及预后生物标志物

近年来，以PD-1/PD-L1抑制剂等免疫检查点抑制剂为代表的免疫疗法进入临床应用，是肺癌治疗领域的一大里程碑事件。免疫疗法很大程度地改善了晚期NSCLC患者的生存预后，且疗效持久。但免疫疗法在未经选择的人群中有效率偏低，如PD-1/PD-L1抑制剂单药用于NSCLC的应答率仅为25%^[86]^。因此寻找合适的分子标志物以筛选优势人群，是实现免疫治疗精准化的关键。

回顾性分析^[87]^提示，携带明确驱动基因变异的NSCLC患者倾向于表现为冷肿瘤的免疫微环境特征，即免疫治疗效果并不理想。CSCO NSCLC诊疗指南(2020)也将肺癌免疫治疗的人群限制为无驱动基因变异的患者^[6]^。IASLC(International Association for the Study of Lung Cancer)专家共识则特别强调，在存在可用药致癌驱动基因变异的肿瘤中，必须禁止前期使用免疫检查点抑制剂，对于晚期患者，在没有获得常规基因组信息(*EGFR*、*ALK*、*ROS1*和*BRAF*)的情况下，不应该使用免疫检查点抑制剂^[87]^。

目前进入临床实践的免疫治疗生物标志物有PD-L1高表达、错配修复基因缺陷(mismatch repair-deficient, dMMR)/微卫星高度不稳定性(high-frequency microsatellite instability, MSI-H)，以及高肿瘤突变负荷(tumor mutation burden-high, TMB-H)。PD-L1高表达(≥50%)是目前NSCLC一线免疫单药治疗唯一获批的分子标志物。但由于肿瘤异质性以及缺乏“金标准”检测方法，加之免疫组化检测方法本身的检测敏感性差异问题，PD-L1检测目前仅在部分肿瘤适应证中作为使用特定药物的临床分子标志物^[87]^。dMMR/MSI-H是获FDA批准、不限组织学类型的免疫治疗生物标志物^[88]^。FDA也已批准了针对MSI-H肿瘤的免疫治疗方案^[88]^。但由于dMMR/MSI-H在肺癌患者中的发生频率较低，其在肺癌免疫治疗中的临床应用价值仍需更多研究探索。

TMB作为预测免疫检查点抑制剂的潜在标志物已逐渐进入临床应用。已有研究表明，组织肿瘤突变负荷高(tissue TMB, tTMB)与NSCLC免疫治疗总体生存率(overall survival, OS)获益有相关性^[89, 90]^，结合PD-L1和TMB可以更好地预测NSCLC患者的免疫治疗疗效^[91]^。基于两项III期研究结果^[92, 93]^，NCCN指南推荐TMB作为新的生物标志物，用于筛选纳武利尤单抗联合伊匹单抗方案和纳武利尤单抗单药治疗方案的优势人群^[5]^。基于II期KEYNOTE-158研究数据，美国FDA近期已批准帕博利珠单抗用于TMB-H(≥10个突变/兆碱基)、既往治疗后病情进展且无满意替代治疗方案的不可手术切除或转移性实体瘤患者^[94]^。此外，为克服组织样本检测的局限性，血液TMB(blood TMB, bTMB)检测的应用逐渐广泛，从POPLAR和OAK研究^[95]^，再到B-FIRST^[96]^、BFAST研究，都显示出bTMB预测免疫治疗疗效的潜力。但由于多家基因检测公司检测TMB时使用的panel所涵盖的基因不同，探针覆盖的基因位置和范围不同(基因全长覆盖或热点区域覆盖)，设定的cut-off值也不同，所以TMB检测目前仍无法标准化，从而限制了其广泛应用于临床。

此外，已有一些研究发现，某些基因变异与免疫治疗效果密切相关，如*KRAS*突变和*TP53*突变的肿瘤可能有更好的免疫疗效^[97]^；抑癌基因*STK11*缺失突变则可能造成免疫治疗的疗效不佳^[98]^。一系列早期研究中显示，一些罕见基因突变可能与肿瘤免疫原性升高有关，如*MUC16*突变、*MET* 14号外显子突变、*BRCA1/2*突变、*POLE*突变、*POLD1*突变和*MSH2*突变^[99]^，但其临床效用仍需大样本的临床研究进一步探索。研究^[100, 101]^发现，有9%-29%的患者在接受免疫检查点抑制剂治疗中出现了超进展(hyperprogression, HP)现象，这部分患者一般预后极差，中位生存时间仅2个月-5个月。目前发现*MDM2/MDM4*扩增、*EGFR*扩增和位于11q13位点的一些基因如*CCND1*、*FGF3*、*FGF4*和*FGF19*等扩增与HP存在相关性，推测这些基因变异可能是预测HP的标志物^[102]^。一系列与免疫治疗预后相关的新兴生物标志物也在持续探索中，如肿瘤浸润淋巴细胞(tumor infiltrating lymphocytes, TIL)、DNA损伤修复(DNA damage repair, DDR)通路基因变异、外泌体中特定蛋白的检测、DNA甲基化、肠道菌群等。

**共识6：结合患者实际临床情况，如需获取免疫治疗相关的分子标志物信息，晚期新发或术后复发的NSCLC患者，建议采用NMPA或FDA批准的NGS产品进行检测，检测包含MSI、tTMB、免疫治疗正负向相关基因和免疫治疗超进展相关基因在内的免疫治疗相关分子标志物。【III级推荐】**

### 3.6 NGS检测平台的选择

为了协助临床合理使用不断革新的治疗策略，理想的NGS检测平台应能够：①单次检测就能发现与临床治疗相关基因的所有类型变异，包括点突变、插入/缺失、拷贝数变异、基因重排；②覆盖的检测范围广泛，可提供TMB、MSI等重要免疫治疗分子标志物；③所需样本少；④检测周期短。为了保证临床实践的准确性，NGS检测结果必须保证检测流程的稳定，包含目标区域富集方法、测序数据量、生物信息学分析流程等一系列可能影响结果准确性的因素^[13]^。

NGS技术包括全基因组测序(whole genome sequencing, WGS)、全外显子组测序(whole exome sequencing, WES)、热点基因变异的NGS小/大panel检测以及全面基因组(comprehensive genomic profiling, CGP)检测等。由于WGS和WES检测覆盖的基因范围过于广泛，纳入了大量与肿瘤不相关的基因，引起检测周期延长、成本上升，进而引起检测深度不足而造成的敏感性下降，加上无法或极少涵盖与肿瘤发生发展或治疗密切相关的融合基因内含子区域，其应用偏向于科研，无法在临床广泛应用^[103]^。

目前国内外获批用于NSCLC的NGS检测方法有热点基因变异NGS检测和全面基因组CGP检测两种。肿瘤热点基因变异NGS panel的检测范围一般仅局限于发生频率较高的特定基因^[104]^，仅检测少数目标基因的特定区域(即已被证实有与高致癌风险相关的区域)^[105]^，并且无法检测所有的基因变异类型^[19]^。我国已获批用于NSCLC患者检测的NGS产品均为热点基因检测^[106]^。CGP检测要求至少覆盖300个以上与肿瘤用药、预后密切相关基因的全外显子区域；覆盖临床意义明确的热点融合基因的内含子区域；包含TMB、MSI检测；并可以准确评估所有基因的拷贝数变化(copy number variation, CNV)。

CGP检测目前已经获得FDA批准的包括FoundationOne^®^CDx和MSK-IMPACT^TM^。FoundationOne^®^CDx是FDA批准的首个NGS泛癌种伴随诊断产品，基于杂交捕获技术，覆盖包括309个基因的全部外显子以及34个常见重排基因的特定内含子，一次检测即可提供324个肿瘤相关基因的四种变异信息，有效测序深度达到500X以上，可检出肿瘤驱动基因的罕见变异形式，同时还能提供TMB和MSI数据^[107]^。FoundationOne^®^CDx是目前唯一一个经过分析验证^[108]^和临床验证^[93, 109, 110]^的泛癌种伴随诊断产品。MSK-IMPACT^TM^是第一个经FDA批准的实验室开发的肿瘤基因组补充诊断产品，MSK-IMPACT^TM^同样也是基于杂交捕获技术，一次检测可提供468个肿瘤相关基因的两种基因变异类型(碱基替换和插入/缺失)。

FDA批准上市应用于临床诊断尤其是伴随诊断的检测方法，对其分析验证及临床验证两方面均有严格要求^[111]^。分析验证主要验证检测技术的方法学以确认分析性能。CGP检测由于其覆盖范围广、分析过程复杂等特点，目前主要以突变类型区分或以临床意义明确的关键基因或位点为代表，对细胞系和临床样本进行检测，与已经获批的PCR、FISH等产品的检测结果进行一致性分析，给出相应的准确性(包含敏感性、特异性、阳性预测值、阴性预测值)和精确度(包括可重复性、可再现性、检出下限)等方法学参数^[112]^。而基于突变在肿瘤发生发展中的复杂生物学机制，在方法学可靠的基础上，更需要关键性的临床验证来确定Cut-off值、临床敏感性与特异性，从而进一步保证检测结果指导下的用药能真正实现患者的临床获益^[10]^。对于一些发展较成熟的突变标志物，例如*EGFR*基因突变，临床验证常采用非劣效性测试进行基因变异检测一致性及相对应药物疗效相关性的回顾性分析。而对于不断涌现的新靶点标志物的检测，尤其是没有对应的PCR、FISH、IHC等传统检测方法获批的分子标志物，前瞻性临床研究数据不可或缺。FoundationOne^®^CDx的TMB检测正是基于其参与的多项II期/III期的免疫治疗药物临床研究^[92-94]^的疗效数据，成为目前唯一一个经过分析验证^[108]^和临床验证^[106-108]^的泛癌种伴随诊断产品。同样，bTMB与Atezolizumab疗效的相关性在通过对POPLAR和OAK试验的回顾性检测得到初步临床验证后，又在NSCLC患者中开展前瞻性的B-FIRST研究及前瞻性BFAST伞式研究来进一步临床验证^[95, 96]^。

目前国内尚无已经获批的CGP检测Panel，但已有几家国内基因检测公司向NMPA提交了肿瘤NGS检测大Panel的注册，相信经过分析验证与临床验证的CGP检测Panel在中国获批后，会为中国肺癌患者带来更广泛的临床获益。

考虑到药物可及性等因素，晚期NSCLC患者的首次分子标志物检测建议使用NMPA或FDA批准的检测方法进行。使用非NGS的检测方法，可能会造成组织样本耗竭进而导致无法再进行后续基因检测，进而使患者错失潜在的靶向治疗机会。纪念斯隆-凯特琳癌症中心的研究发现^[27]^，在非NGS检测提示驱动基因变异阴性的NSCLC患者中，有34%的患者在组织耗竭的情况下不适合或拒绝再次穿刺活检。而CGP可节省序贯单基因测序或热点基因变异检测可能带来的样本/时间/成本消耗^[24]^。由此我们认为，在患者有意愿获取最为全面的基因变异信息的情况下，晚期NSCLC患者首次基因检测应使用经过NMPA或FDA批准的针对肿瘤基因的CGP NGS检测方法，这一点对于肿瘤样本量少的患者尤其重要。但同时也应当了解NGS的应用局限性，如受样本取材或探针设计等因素影响或限制，NGS在某些变异类型如拷贝数扩增的检测方面存在一定的局限性。对于NGS方法可能无法准确检出的部分基因变异，应考虑采用可靠的单基因检测方法对NGS检出的变异进行验证^[113]^。

**共识7：在条件允许的情况下，如患者有意愿获取最为全面的基因变异信息，晚期新发或术后复发的NSCLC患者，首次基因检测可以自行选择NMPA或FDA批准的CGP NGS检测产品，在全面了解肿瘤基因组信息的基础上制订一线治疗方案。【II级推荐】**

### 3.7 NGS液体活检可以作为组织检测的重要补充

肿瘤组织样本的基因检测在临床实际应用中仍存在诸多限制，包括取样操作的有创性、组织样本有限或耗竭、肿瘤内部异质性而导致采样偏差^[114, 115]^、无法针对所有转移灶取样^[116]^、可能因肿瘤位置特殊而无法取样^[115]^、无法动态监测肿瘤变化^[117]^等。

近年来，液体活检技术的发展为肿瘤患者提供了一种非侵入性取材的检测路径。液体活检是指标志物的收集和分析来自于各种体液，如血液、尿、痰、脑脊液和胸腔积液等。这些标志物可以是循环肿瘤细胞CTC、cfDNAs、cfRNAs、循环外泌体、蛋白质及其他代谢物等^[118]^。

目前从血清上清液或者血浆中提取ctDNA的液体活检技术相对成熟^[119]^，且大量的临床研究证实了血液样本中的ctDNA检测可评估肿瘤的复发、耐药和转移^[120]^。有研究^[121, 122]^证实，治疗后ctDNA是否清零可以评估疗效，ctDNA的上升也可以提前预示病情进展。此外，为克服组织样本检测的局限性，bTMB检测的应用逐渐广泛，从POPLAR和OAK研究^[95]^，再到B-FIRST^[96]^、BFAST研究，都显示出bTMB预测免疫治疗疗效的潜力。相比组织样本检测，血液样本检测可简化采样流程；可多次采样，进行实时监控；减少采样偏差；降低取样创伤和患者死亡率^[123]^。

除了血液ctDNA检测外，使用脑脊液(cerebrospinal fluid, CSF)和胸腔积液(malignant pleural effusion, MPE)进行液体活检的研究也越来越多。有研究^[124]^表明，对于脑转移患者，血浆ctDNA的检出率仅有50%左右，CSF中非肿瘤来源的DNA含量低，更容易检测到突变。尤其是在脑膜转移的患者中，CSF ctDNA体现出比血液ctDNA更优的检测性能^[125, 126]^。但由于CSF的获取方式为微创手术，如何筛选应进行CSF检测的人群也是临床必须考虑的问题。基于最大转移灶直径和全部转移灶距离中枢神经距离的评分系统，可以将非脑膜转移的脑转移患者的脑脊液CSF ctDNA阳性检出率从59.09%提高至81.81%^[127]^。此外，也有报道^[125-129]^指出脑脊液中可发现更多获得性耐药基因突变。胸腔积液也是可用于NGS液体活检的可靠标本^[130, 131]^。有研究者^[132]^认为，在对于胸膜和腹膜受累患者进行基因突变检测时，更靠近转移部位的液体(胸腔积液和胸腹水)要优于血液，并且可以在常规临床实践中对这些“非血液”液体进行基因检测，以选择具有特定转移部位的癌症患者的抗肿瘤治疗药物。值得注意的是，由于目前其他液体标本仍缺乏大样本量的基因变异类型与临床靶向治疗疗效相关性的前瞻性研究，临床中广泛应用的ctDNA检测仍然集中于血液标本。

尽管液体活检有利于克服肿瘤异质性并实现基因变异图谱实时监测，但由于体液中ctDNA含量极低，同时具有高度片段化、半衰期较短的特点，并且ctDNA的释放受肿瘤类型、分期、转移灶的部位等多种因素影响^[133, 134]^，限制了其在临床上的广泛应用。ctDNA的特点也对样本处理提出了更为严格的要求，并对灵敏度和特异性的要求更高，否则可能会引起假阴性的结果。

虽然目前国内尚无临床获得NMPA或FDA批准的用于ctDNA检测NGS产品，也无成熟的液体ctDNA检测的行业标准。但2019年3月，第一款肿瘤NGS液体活检产品Personal Genome Diagnostics(PGDx)公司的ElioTM Plasma Resolve经欧盟批准上市，弥补了肿瘤基因检测市场上仅有NGS组织活检产品的不足，同时也宣告NGS液体活检正式进入临床应用。2020年8月7日，FDA批准了第一个液体活检伴随诊断试剂盒，来自Guardant Health公司的包含55个基因的Guardant 360 CDx用于识别携带*EGFR*基因突变的转移性NSCLC患者，以帮助确定可以从奥希替尼治疗中获益的患者。随后，在8月26日，FDA批准了首个基于液体活检的CGP伴随诊断产品Foundation Medicine公司的FoundationOne^®^liquid CDx(F1LCDx)。F1LCDx经过严格的技术验证和临床验证，可以全面准确识别324个基因的四种变异类型(点突变、插入/缺失、拷贝数变异和基因重排)，且是首个同时提供免疫治疗相关生物标志物bMSI/bTMB以及评估ctDNA含量的伴随诊断产品。

由于国内目前暂未获批基于液体活检的NGS产品，CSCO NSCLC诊疗指南(2020)仍建议针对NSCLC患者的基因检测，首选组织检测，只有在组织标本量不足或者组织不可及的情况下，可以选择液体活检作为补充手段。

**共识8：晚期新发或术后复发的NSCLC患者，首次基因检测组织样本不足或组织检测失败时，经NMPA或FDA批准的液体活检检测产品可作为辅助或补充。【II级推荐】**

**对于脑转移患者(包含脑膜转移)，有证据表明脑脊液ctDNA体现出比血液ctDNA更好的检测性能，在条件允许的情况下，优先使用脑脊液进行液体活检。【III级推荐】**

**有证据表明，胸腔积液ctDNA体现出比血液ctDNA更好的检测性能，在条件允许的情况下，在胸腔积液沉渣中病理分析检出肿瘤细胞的前提下，优先使用胸腔积液进行液体活检。【III级推荐】**

## 4 靶向治疗耐药NSCLC患者进行基因检测的共识意见

在肺癌的分子靶向药物治疗中，耐药是临床难以避免的挑战，了解耐药机制是合理选择后续治疗方案的前提。NSCLC靶向治疗的耐药机制复杂，包括驱动基因的二次突变、旁路或下游通路激活、组织或表型转化等。因此，对靶向治疗耐药的患者再次进行广泛的分子标志物检测有助于全面分析耐药机制，并指导后续用药。

### 4.1 NSCLC常见驱动基因靶向治疗耐药后检测

EGFR-TKI耐药机制多样，不同药物也存在差异。目前最常报道的一代/二代EGFR-TKI耐药相关突变为*EGFR* T790M突变。对一代/二代EGFR-TKI耐药患者，CSCO NSCLC诊疗指南(2020)建议再次活检时应检测*EGFR* T790M，T790M突变阳性患者可使用三代EGFR-TKI奥希替尼治疗^[6]^。*MET*或*ERBB2*扩增也与EGFR-TKI耐药相关，NCCN指南将其纳入EGFR-TKI耐药基因检测内容^[5]^。基于广泛的分子标志物检测，更多EGFR-TKI耐药相关的基因变异不断被发现，如基于FoundationOne^®^CDx检测发现的*RET*重排共突变(与一代和二代EGFR-TKI耐药相关)^[135]^和*FGFR3-TACC3*融合突变^[136]^，基于MSK-IMPACT TM检测发现的*BRAF*融合、*FGFR3*融合、*YES1*扩增、*KEAP1*丢失和*MTOR* E2419K突变^[137]^。

一代和二代ALK-TKI药物的耐药基因变异存在差异。一代ALK-TKI克唑替尼治疗后发生*ALK*耐药突变的发生概率为20%，最常见的耐药突变为L1196M(7%)和G1269A(4%)。二代ALK-TKI最常见的耐药突变是G1202R：塞瑞替尼耐药患者中最常见的耐药突变为G1202R(21%)、F1174C/L(16.7%)和C1156Y(8%)；阿来替尼耐药患者中最常见的耐药突变为G1202R(29%)，其他突变包括I1171T/S(12%)、V1180L(6%)、L1196M(6%)；Brigatinib耐药患者中最常见的突变为G1202R^[32]^。ALK-TKI耐药患者检出*ALK*耐药突变，说明肿瘤仍为ALK依赖，使用其他ALK抑制剂可能有效。根据ALK-TKI对各种耐药细胞株作用研究表明，三代ALK-TKI劳拉替尼对绝大多数已知*ALK*耐药型突变有效^[32]^，但劳拉替尼对一代和二代ALK-TKI耐药NSCLC的疗效不同。克唑替尼治疗后耐药且未使用过其他ALK抑制剂的患者，是否出现*ALK*耐药突变并不影响劳拉替尼的疗效，这可能与克唑替尼是一个不完全ALK通路抑制剂相关；而对于二代ALK-TKI治疗后耐药患者，未检出*ALK*耐药突变，使用三代ALK抑制剂就很难获益^[138]^。

*ROS1*重排阳性NSCLC患者接受克唑替尼治疗后耐药的机制目前尚不完全明确，已有的研究^[139]^证据提示一系列点突变(最常检出的是G2032R，其他还有D2033N、L2026M、S1986Y/F、L2155S)、旁路激活(如KIT和EGFR通路激活)和下游通路异常(如RAS/RAF/MEK通路、PI3K通路、JAK/STAT通路)与克唑替尼耐药相关。旁路激活、下游通路异常导致的耐药可选择对应的靶向联合治疗^[140]^或化疗/靶向联合免疫方案^[141]^，第三代ALK-TKI劳拉替尼也显示对旁路激活相关的克唑替尼耐药肿瘤有效^[142]^。针对点突变导致的耐药，可根据具体的点突变选择相应的靶向药物，如对携带G2032R的NSCLC有潜在治疗作用的靶向药物有卡博替尼和Ropotrectinib^[118]^。

### 4.2 NSCLC少见驱动基因靶向治疗耐药后检测

除上述NSCLC中常见驱动基因TKI耐药机制外，近几年针对少见靶点TKI潜在耐药机制的研究也有一些报道，具体如下。

关于靶向*BRAF* V600E突变治疗的耐药机制的研究比较有限。3例*BRAF* V600E的NSCLC患者分别在接受达拉非尼单药或达拉非尼联合曲美替尼治疗耐药后，报道了获得性的*KRAS* G12D、*KRAS* G12V和*NRAS* Q61K突变，提示可能是耐药机制^[143-145]^。基于MATCH-R(matching resistance)研究患者数据的文章^[146]^报道了11例针对BRAF V600E靶向治疗后的潜在耐药机制：4例患者分别检测到了*MEK1* K57N、*PTEN* N329fs、*NRAS* Q61R以及*KRAS* Q61R突变。综上所述，靶向*BRAF* V600E治疗的潜在耐药机制主要涉及上下游*RAS*/*MEK*基因的突变以及旁路影响(PI3K通路)。

关于靶向*NTRK*基因融合治疗的耐药研究集中在临床前证据以及其他癌种个例报道，肺癌作为低频癌种尚未有相关报道。相关耐药机制多为*NTRK*激酶区的获得性耐药突变，包括*TRKA* G595R/F589L/A608D/G667C、*TRKC* G623R/G696A，目前并未见报道发生在*TRKB*上的耐药突变^[41]^。针对这些耐药突变的二代NTRK-TKI，如LOXO-195^[147]^和TPX-0005^[148]^正在临床试验探索阶段。除*NTRK*耐药突变外，NTRK-TKI也可能存在旁路激活耐药机制，临床前研究^[149]^表明*IGF1R*旁路激活是潜在耐药机制；STARTRK-2研究的后续分析也报道了恩曲替尼耐药患者中获得性的*BRAF* V600E和*KRAS* G12D突变，提示与MAPK通路的潜在相关性^[150]^。

*MET*靶向治疗耐药机制的研究主要针对*MET* 14号外显子跳跃突变。多篇案例^[151, 152]^报道了克唑替尼治疗*MET* 14号外显子跳跃突变NSCLC患者进展后的突变位点，包括*MET* D1228X和Y1230X。2019 ASCO会议上报道了更加全面的*MET*靶向治疗潜在耐药机制^[153]^，包括在靶突变(*HGF*扩增、*MET* D1228X/Y1230X/G1163R/L1195V/H1049Y以及*MET* 14号外显子扩增)和旁路激活(*EGFR*扩增、*ERBB2*扩增^[154]^、*KRAS*扩增、*KRAS* G12S/G13V以及*RASA1* S724*)，其中激酶区突变为MET抑制剂耐药的主要原因。上述这些研究大部分是基于克唑替尼治疗耐药患者的研究，这些耐药位点是否对新一代选择性MET抑制剂敏感，还有待临床探索。

针对NSCLC中*ERBB2*以及*RET*靶向治疗耐药机制的研究较少。一项体外研究^[155]^报道了C805S突变是携带*ERBB2* 20号外显子突变患者接受靶向治疗后的获得性耐药机制。另一项针对多例*ERBB2*突变NSCLC患者的回顾性分析^[156]^提示*PIK3CA*突变(R88Q)以及*ERBB2*扩增是潜在耐药机制。近期，一项体外研究^[157]^发现一些*RET*突变(L730/E732/V738/V804/Y806/A807/G810)与髓样甲状腺癌患者Cabozantinib、Lenvatinib、Vandetanib耐药相关，到目前为止，这些突变还没有在*RET*融合或*RET*突变癌症患者的肿瘤样本中得到证实。近期一个病例^[158]^报道了*RET*融合的NSCLC患者在Vandetanib耐药时出现二次*RET*突变(S904F)。另一个*RET*融合NSCLC的案例^[159]^描述了V804M突变对Vandetani耐药的影响。这些结果表明，*RET*激酶区突变可能是产生TKI耐药的原因。

### 4.3 免疫治疗耐药后检测

由于免疫治疗的耐药机制尚不清晰，并且尚无免疫治疗后产生可靶向治疗耐药位点的文献报道，因此本共识并未对免疫治疗耐药后患者进行相关NGS检测的推荐。

**共识9：对于靶向治疗耐药后的患者，为了更好地指导后续治疗方案的选择，建议使用NMPA或FDA批准的CGP NGS检测产品再次进行检测。【II级推荐】**

对于不能获取肿瘤标本的耐药患者，CSCO NSCLC诊疗指南(2020)建议进行基于血液样本的cf/ctDNA检测^[6]^。液体检测因无创和操作便捷，可动态监测治疗效果，及早发现耐药。疾病进展时，cfDNA分析可有效识别导致耐药的新发基因变异。如通过ctDNA检测出了EGFR-TKI的新型获得性耐药突变*FGFR3-TACC3*融合，提示基因激酶区的融合可能是EGFR-TKI的耐药机制^[136]^。FLAURA研究中，通过ctDNA检测到最常见的奥希替尼耐药相关突变为*MET*扩增和*EGFR* C797S突变，其他突变包括*HER2* 扩增、*PIK3CA*和*RAS*突变^[160]^。此外，通过ctDNA检测还发现了ALK抑制剂的新型耐药突变^[161-163]^。

**共识10：靶向治疗耐药后优先选择再次活检，如无法再次进行组织活检或活检组织样本不足时，可使用经NMPA或FDA批准的CGP NGS液体活检检测产品进行补充检测。【II级推荐】**

## 5 NGS检测中标本类型和检测流程的规范

样本质控对高质量基因检测结果至关重要。适合临床NGS分析的样本类型优选新鲜组织标本，也可选用甲醛固定-石蜡包埋样本(formalin-fixed paraffin-embedded, FFPE)、肿瘤细胞学样本及血浆(游离DNA/RNA)等^[11]^。

NGS检测样本的采集方法包括手术切除样本、活检小样本、细针抽吸组织(细胞块)和积液脱落细胞学(细胞块)。对于未接受过靶向/免疫药物治疗的患者，NGS检测应首选经病理评估的组织样本，优先使用手术切除样本，也可选择活检样本(选择肿瘤含量最高或肿瘤最集中部位)，再次检测可选取液体活检样本进行动态监测^[11, 13]^。接受过靶向/免疫药物治疗的患者，应尽可能使用治疗后的样本，以寻找治疗后新出现的耐药突变。

各个NGS检测实验室对不同样本(新鲜组织标本、FFPE样本、细胞学样本和体液/血液)的采集和处理需制订严格的标准操作规程(standard operating procedure, SOP)。样本采集最好采用一次性材料。制备不同患者病理切片样本时，需更换新刀片，并清除操作器皿或切片机上先前样本的残留。

手术和活检的新鲜组织：获取的样本应含有丰富的肿瘤细胞，浸润性肿瘤组织含有一定的淋巴细胞。手术取样后用生理盐水冲洗至组织表面没有明显血迹，避免血液中的gDNA污染。手术采集的肿块组织不低于50 mg(豌豆粒大小)。穿刺样本应≥2条，长度≥0.5 cm。新鲜组织样本的理想保存方法是迅速置于液氮中，可保存于液氮罐或-80 ℃冰箱保存，这一过程应在组织离体后30 min内完成，以防止RNA等核酸降解。也可保存在样本保护剂中，并尽早转移至-80 ℃冰箱保存。可采用冷冻切片染色评估样本中的肿瘤细胞含量。

FFPE：按病理规范要求取材，尽量避免肿瘤细胞过少或未检测到的肿瘤组织、病变中心广泛纤维化的肿瘤、黏液产生过高的肿瘤、周围炎症严重的肿瘤、含坏死组织过多的肿瘤、含钙化灶并脱钙处理过的肿瘤。务必在组织离体后30 min内浸入足量的4%甲醛溶液中固定(液体与组织大小比例为10:1)，避免使用酸性及含有重金属离子的固定液。大标本切开后充分固定12 h-48 h，不超过72 h；小活检标本可固定6 h-12 h。开展NGS检测前应进行HE染色，评估肿瘤细胞的含量。一般情况下，适合于NGS检测的组织样本中肿瘤含量建议应达到20%以上，最佳含量为30%以上。肿瘤细胞体积至少达到0.2 mm^3^。组织样本抽提获得的DNA量应达50 ng以上。若组织样本中肿瘤细胞含量较低，仍需进行NGS检测的话，应尽量采用显微切割技术富集肿瘤细胞，并在报告中提示样本局限性。

细胞学样本：符合病理质控或通过肿瘤细胞富集处理后符合要求的标本，可直接抽提核酸，也可以制备成FFPE细胞学蜡块进行分析。肿瘤科医师在申请胸水标本的基因检测时，必须同时进行液基细胞学检测来进行病理评估。体腔积液标本也可提取无细胞上清标本中的ctDNA进行检测。

血浆或血液样本：采集外周血提取血浆游离DNA进行检测，取样时应使用一次性的含乙二胺四乙酸(ethylenediaminetetraacetic acid, EDTA)的抗凝真空采血管，根据检测平台的要求采集10 mL全血，2 h内分离血浆，提取游离DNA后，保存于-80 ℃冰箱中，并避免反复冻融。如外周血需长时间运输，建议用商品化的游离DNA样本专用保存管，在常温条件下可稳定保存3 d-7 d。

样本运送过程要确保各类样本运送安全、无污染、无降解。石蜡样本及血检样本可常温运输，核酸样本需在4 ℃或冷冻条件下运输。

**共识11：NSCLC的NGS检测样本的采集处理和保存应符合规范要求。未接受过靶向/免疫药物治疗的患者，NGS检测应首选经病理评估的组织样本。接受过靶向/免疫药物治疗的患者，应尽可能使用治疗后的样本。【I级推荐】**

NGS检测的全部流程主要包括实验室操作和生物信息学分析两部分。实验室操作部分包括从符合送检要求的样本中提取DNA、基于杂交捕获或扩增子建库方法进行文库制备、目标区域富集、测序等；生物信息分析部分是对于不同类型变异(包括碱基替换、插入/缺失、拷贝数改变和基因重排)采用特定生物信息学分析方法进行分析并结合药物注释信息给出临床报告。送检样本及全部测序和分析报告流程应符合《临床分子病理学实验室二代基因检测专家共识》、《二代测序技术在肿瘤精准医学诊疗中的应用专家共识》等共识基本要求^[11, 13]^，充分关注试剂管理、样本的质控/质量保证、检测环境、人员资质和熟练程度，NGS技术参数标准(包括文库构建、测序流程、质控等)等环节，配备独立的质量控制程序。

**共识12：NSCLC的NGS检测流程应符合规范要求，配备完善的标准操作流程和独立的质量控制程序。【I级推荐】**

## 6 NGS检测报告的临床解读

NGS检测结果转化为患者的个体化精准诊疗方案，需要基于多个分子标志物的临床或临床前分子生物学证据，并结合患者诊疗史准确判断证据优先级，个性化地解读基因检测报告。基于临床信息的传统肿瘤多学科协作治疗模式(multidisciplinary team, MDT)可能无法满足肺癌临床日益增长的基因检测报告解读需求，尤其是对于耐药后有多种潜在治疗方案的患者，需要结合患者诊疗史对不断更新的证据等级进行个性化排序。由此看来，组建由肿瘤科医师、病理科医师、分子生物学专家、生物信息学专家等多个专业领域专家组成的肺癌分子肿瘤专家委员会(molecular tumor boards, MTB)，讨论分子生物学相关证据，优化患者的个体化诊疗方案，并建立基于分子标志物检测的临床治疗路径或许是更优的解决方案。进入NSCLC-MTB的肿瘤科医师需要基于患者的临床病理学信息发起基因检测需求，熟悉基因变异信息对应的患病风险、靶向治疗、预后疗效等临床应用价值，并对患者基于分子检测的治疗方案进行跟踪随访，以确认分子标志物阳性患者的真实世界疗效；病理科医师能够评估患者的肿瘤标本特征并提供符合检测需求的送检样本，如在院内病理科进行NGS检测，还需要病理科医师对于检测流程进行严格的质量控制，以保证检测结果的准确可信；分子生物学及生物信息学专家主要负责分析基因变异和进行药物相关性注释，及时更新数据库的证据等级以便进行更准确的基因变异与药物相关性注释，并协助临床试验设计和数据收集。如有需要，NSCLC-MTB还应包括肿瘤放射科、肿瘤外科、遗传学和药理学等其他专科领域的专家。

**共识13：二代测序技术为NSCLC患者带来了更多更全面的基因突变信息，倡导各单位组建NSCLC-MTB，以正确解读基因检测的结果，制定个体化临床治疗决策。【II级推荐】**

## 7 展望

二代测序技术的全面应用，将为NSCLC患者个体化临床诊治奠定基础(表 4-表 6)。未来可以通过对患者临床诊疗信息、基因检测结果、后续治疗方案的全程管理，建立NSCLC患者个体化精准治疗平台，同时积累真实世界数据，不断丰富和完善我国NSCLC患者的个体化诊疗全景图。此外，重视肿瘤临床医师与病理科医生、分子生物学及生物信息学专家的合作，构建分子肿瘤专家委员会的新型多学科诊疗模式也是肿瘤临床和研究的重要发展方向。

**4:** 二代测序技术在NSCLC中的临床应用中国专家共识要点 Summary of recommendations

序号	专家共识推荐要点	推荐级别
NGS检测的适用人群		
共识1	推荐所有病理诊断为肺腺癌、含有腺癌成分的肺癌以及不能分型的晚期新发或术后复发的NSCLC患者常规进行基因检测。	I级推荐
	对经小标本活检诊断为含有腺癌成分或具有腺癌分化的混合型鳞癌，以及年轻或不吸烟/少吸烟肺鳞癌患者，也推荐进行基因检测。	II级推荐
共识2	针对敏感型突变发生率高的NSCLC患者(见“共识1”)，常规基因检测结果为阴性时，建议使用中国NMPA或美国FDA批准的NGS产品进行复检。	III级推荐
晚期新发或术后复发的NSCLC患者首次进行基因检测的共识意见		
共识3	针对晚期新发或术后复发的NSCLC患者，首次检测建议采用NMPA批准的检测产品，检测至少包括NSCLC常见驱动基因：*EGFR*突变(应涵盖18号、19号、20号、21号外显子)以及*ALK*融合、*ROS1*融合。	I级推荐
共识4	针对晚期新发或术后复发的NSCLC患者，结合患者实际临床情况，如需获得更多的潜在靶点信息，首次检测建议采用NMPA或FDA批准的检测产品，检测包括*BRAF* V600E、*KRAS*(如G12C等)、*NTRK1/2/3*融合、*MET* 14号外显子跳跃突变和*MET*扩增、*ERBB2* 20号外显子插入、RET融合等少见驱动基因变异。	II级推荐
共识5	共识3和共识4中基因检测均为阴性的NSCLC患者再次检测时，建议采用NMPA或FDA批准的NGS产品，检测包含*EGFR*罕见变异形式(包括激酶区重复和融合)、*BRAF*罕见变异形式(包括激酶区重复和融合)、*MET*罕见变异形式(包括激酶区重复和融合)、*ERBB2*融合等罕见变异形式。	III级推荐
共识6	结合患者实际临床情况，如需获取免疫治疗相关的分子标志物信息，晚期新发或术后复发的NSCLC患者，建议采用NMPA或FDA批准的NGS产品进行检测，检测包含MSI、tTMB、免疫治疗正负向相关基因和免疫治疗超进展相关基因在内的免疫治疗相关分子标志物。	III级推荐
共识7	在条件允许的情况下，如患者有意愿获取最为全面的基因变异信息，晚期新发或术后复发的NSCLC患者，首次基因检测可以自行选择NMPA或FDA批准的CGP NGS检测产品，在全面了解肿瘤基因组信息的基础上制订一线治疗方案。	II级推荐
共识8	晚期新发或术后复发的NSCLC患者，首次基因检测组织样本不足或组织检测失败时，经NMPA或FDA批准的液体活检检测产品可作为辅助或补充。	II级推荐
	对于脑转移患者(包含脑膜转移)，有证据表明脑脊液ctDNA体现出比血液ctDNA更好的检测性能，在条件允许的情况下，优先使用脑脊液进行液体活检。	III级推荐
	有证据表明，胸腔积液ctDNA体现出比血液ctDNA更好的检测性能，在条件允许的情况下，在胸腔积液沉渣中病理分析检出肿瘤细胞的前提下，优先使用胸腔积液进行液体活检。	III级推荐
靶向治疗耐药后的NSCLC患者进行检测的共识意见		
共识9	对于靶向治疗耐药后的患者，为了更好的指导后续治疗方案的选择，建议使用NMPA或FDA批准的CGP NGS检测产品再次进行检测。	II级推荐
共识10	靶向治疗耐药后优先选择再次活检，如无法再次进行组织活检或活检组织样本不足时，可使用经NMPA和FDA批准的CGP NGS液体活检检测产品进行补充检测。	II级推荐
NGS检测的标本类型和检测流程的规范		
共识11	NSCLC的NGS检测样本的采集处理和保存应符合规范要求。未接受过靶向/免疫药物治疗的患者，NGS检测应首选经病理评估的组织样本。接受过靶向/免疫药物治疗的患者，应尽可能使用治疗后的样本。	I级推荐
共识12	NSCLC的NGS检测流程应符合规范要求，配备完善的标准操作流程和独立的质量控制程序。	I级推荐
NGS检测报告的临床解读		
共识13	二代测序技术为NSCLC患者带来了更多更全面的基因突变信息，倡导各单位组建NSCLC-MTB，以正确解读基因检测的结果，制订个体化临床治疗决策。	II级推荐
本共识中涉及的推荐等级和证据水平参照2020版CSCO NSCLC诊疗指南(表 5-表 6)^[6]^；NMPA：国家药品监督管理局；FDA：食品药品监督管理局；NGS：二代基因测序；CGP：全面基因组；MSI：微卫星不稳定性；tTMB：组织肿瘤突变负荷。		

**5:** 共识证据类别 Evidence level

证据特征			专家共识度
类别	水平	来源	
1A	高	严谨的*meta*分析，大型随机对照临床研究	一致共识(支持意见≥80%)
1B	高	严谨的*meta*分析，大型随机对照临床研究	基本一致共识，但争议小(支持意见60%-80%)
2A	稍低	一般质量的*meta*分析，小型随机对照研究，设计良好的大型回顾性研究，病例-对照研究	一致共识(支持意见≥80%)
2B	稍低	一般质量的*meta*分析，小型随机对照研究，设计良好的大型回顾性亚久，病例-对照研究	基本一致共识，但争议小(支持意见60%-80%)
3	低	非对照的单臂临床研究，病例报告，专家观点	无共识，且争议大(支持意见 < 60%)

**6:** 共识推荐等级 Recommendation level

推荐等级	证据类别标准
I级推荐	- 1A类证据 -部分专家共识度高且在中国可及性好的2A类证据
II级推荐	- 1B类证据 -部分专家共识度稍低或在中国可及性不太好的2A类证据
III级推荐	- 2B类证据 - 3类证据
不推荐/反对	-已有充分证据证明不能使患者获益，甚至导致患者伤害的药物或医疗技术，专家组具有一致共识。可以是任何类别的证据。

**Table:** 本共识专家组成员

	医院	姓名
执笔专家	同济大学附属上海市肺科医院	周彩存
执笔专家	中国医学科学院肿瘤医院	王洁
执笔专家	吉林省肿瘤医院肿	程颖
执笔专家	上海交通大学附属胸科医院	韩宝惠
执笔专家	四川大学华西医院	卢铀
执笔专家	天津医科大学肿瘤医院	王长利
执笔专家	山东省肿瘤医院	王哲海
执笔专家	浙江省肿瘤医院肿瘤	范云
执笔专家	北京大学肿瘤医院	林冬梅
执笔专家	同济大学附属上海市肺科医院	任胜祥
执笔专家	中国人民解放军东部战区总医院	宋勇
执笔专家	华中科技大学同济医学院附属协和医院	伍钢
执笔专家	空军军医大学第一附属医院	张艰
执笔专家	广东省人民医院	张绪超
参与专家	中山大学肿瘤防治中心	张力
参与专家	复旦大学附属肿瘤医院	常建华
参与专家	北京大学肿瘤医院	陈克能
参与专家	浙江省肿瘤医院	陈明
参与专家	上海交通大学附属胸科医院	傅小龙
参与专家	中国医学科学院肿瘤医院	高树庚
参与专家	云南省肿瘤医院	黄云超
参与专家	中国人民解放军总医院	焦顺昌
参与专家	上海交通大学附属胸科医院	陆舜
参与专家	河南省肿瘤医院	马智勇
参与专家	中国科学技术大学附属第一医院	潘跃银
参与专家	江苏省人民医院	束永前
参与专家	武汉大学人民医院	宋启斌
参与专家	苏州大学附属第一医院	陶敏
参与专家	中国医学科学院肿瘤医院深圳医院	王绿化
参与专家	北京大学肿瘤医院	王子平
参与专家	武汉大学中南医院	谢丛华
参与专家	同济大学附属上海市肺科医院	许亚萍
参与专家	北京大学人民医院	杨帆
参与专家	广东省人民医院	杨衿记
参与专家	山东省肿瘤医院	袁双虎
参与专家	中山大学肿瘤防治中心	张兰军
参与专家	天津医科大学肿瘤医院	赵路军
参与专家	陆军医科大学新桥医院	朱波
参与专家	中日友好医院	朱广迎
参与专家	中国医学科学院肿瘤医院	段建春
参与专家	同济大学附属上海市肺科医院	何雅億
参与专家	中国医学科学院肿瘤医院	王志杰

## References

[b1] 1Globocan 2018 (IARC), Available from: http://gco.iarc.fr/. Accessed August 31, 2020.

[2] Chinese Medical Association, Oncology Society of Chinese Medical Association, Chinese Medical Association Publishing House (2018). Chinese medical association guidelines for clinical diagnosis and treatment of lung cancer (Edition 2018). Zhong Liu Yan Jiu Yu Lin Chuang.

[3] Zeng HM, Chen WQ, Zheng RS (2018). Changing cancer survival in china during 2003-15: A pooled analysis of 17 population-based cancer registries. Lancet Glob Health.

[b4] 4Surveillance, Epidemiology, and End Results (SEER) Program; National Cancer Institute. SEER Cancer Statistics Review, 1975-2009 (Vintage 2009 Populations), Updated August 20, 2012. Lung and Bronchus. Available from: http://seer.cancer.gov/csr/1975_2009_pops09/results_merged/sect_15_lung_bronchus.pdf. Accessed August 31, 2020.

[b5] 5National Comprehensive Cancer Network. NCCN Clinical Practice Guidelines in Oncology: non-small cell lung cancer, Version 6. 2020. Available from: http://www.nccn.org/default.aspx. Accessed August 31, 2020.

[6] 中国临床肿瘤学会(CSCO) (2020). NSCLC诊疗指南(2020版).

[7] Lindeman NI, Cagle PT, Aisner DL (2018). Updated molecular testing guideline for the selection of lung cancer patients for treatment with targeted tyrosine kinase inhibitors: Guideline from the College of American Pathologists, the International Association for the Study of Lung Cancer, and the Association for Molecular Pathology. J Thorac Oncol.

[8] Kalemkerian GP, Narula N, KennedyEB (2018). Molecular testing guideline for the selection of patients with lung cancer for treatment with targeted tyrosine kinase inhibitors: American Society of Clinical Oncology Endorsement of the College of American Pathologists/International Association for the Study of Lung Cancer/Association for Molecular Pathology Clinical Practice Guideline Update. J Clin Oncol.

[9] Matthijs G, Souche E, Alders M (2016). Guidelines for diagnostic next-generation sequencing. Eur J Hum Genet.

[10] Jennings LJ, Arcila ME, Corless C (2017). Guidelines for validation of next-generation sequencing based oncology panels: a joint consensus recommendation of the Association for Molecular Pathology and College of American Pathologists. J Mol Diagn.

[11] Preparation group of Expert Consensus on Second-generation Gene Sequencing Detection in Clinical Molecular Pathology Laboratory (2017). Expert consensus on second-generation gene sequencing detection in clinical molecular pathology laboratories. Zhonghua Bing Li Xue Za Zi.

[12] Huang H, Shen YP, Gu WH (2018). Discussion on the clinical genetic testing report and the consensus of gene testing industry. Zhonghua Yi Xue Yi Chuan Xue Za Zhi.

[13] Tumor Biomarker Committee of the Chinese Society of Clinical Oncology, China Actionable Genome Consortium (2018). Application of next-generation sequencing technology to precision medicine in cancer: joint consensus of the Tumor Biomarker Committee of the Chinese Society of Clinical Oncology. Zhonghua Yi Xue Za Zhi.

[14] Hematology Oncology Committee of China Anti-Cancer Association, Chinese Society of Hematology Chinese Medical Association, Chinese Society of Pathology Chinese Medical Association (2018). Expert Consensus on the Application of Next Generation Sequencing in Hematological Neoplasms (2018). Zhonghua Xue Ye Xue Za Zhi.

[b15] 15Catalogue of Somatic Mutations in Cancer (COSMIC). Available from: https://cancer.sanger.ac.uk/cosmic/signatures. Accessed August 31, 2020.

[16] Paik PK, Varghese AM, Sima CS (2012). Response to erlotinib in patients with *EGFR* mutant advanced non-small cell lung cancers with a squamous or squamous-like component. Mol Cancer Ther.

[17] Wong DW, Leung EL, So KK (2009). The *EML4-ALK* fusion gene is involved in various histologic types of lung cancers from nonsmokers with wild-type EGFR and KRAS. Cancer.

[18] Barbany G, Arthur C, Liedén A (2019). Cell free tumour DNA testing for early detection of cancer-a potential future tool. J Intern Med.

[19] Schrock AB, Frampton GM, Herndon D (2016). Comprehensive Genomic Profiling Identifies Frequent Drug-Sensitive EGFR Exon 19 Deletions in NSCLC not Identified by Prior Molecular Testing. Clin Cancer Res.

[20] Frampton GM, Ali SM, Rosenzweig M (2015). Activation of MET via diverse exon 14 splicing alterations occurs in multiple tumor types and confers clinical sensitivity to MET inhibitors. Cancer Discov.

[21] Matos LL, Trufelli DC, de Matos MG (2010). Immunohistochemistry as an important tool in biomarkers detection and clinical practice. Biomark Insights.

[22] Ross JS, Gay LM, Wang K (2016). Nonamplification *ERBB2* genomic alterations in 5, 605 cases of recurrent and metastatic breast cancer: an emerging opportunity for Anti-HER2 targeted therapies. Cancer.

[b23] 23Melissa Conrad Stöppler. Polymerase Chain Reaction (PCR): Steps in This DNA Test. Available from: https://www.medicinenet.com/pcr_polymerase_chain_reaction/article.htm#what_is_pcr_polymerase_chain_reaction.

[24] Pennell NA, Arcila ME, Gandara DR (2019). Biomarker testing for patients with advanced non-small cell lung cancer: real-world issues and tough choices. Am Soc Clin Oncol Educ Book.

[25] Rozenblum AB, Ilouze M, Dudnik E (2017). Clinical impact of hybrid capture-based next-generation sequencing on changes in treatment decisions in lung cancer. J Thorac Oncol.

[26] Ali SM, Hensing T, Schrock AB (2016). Comprehensive genomic profiling identifies a subset of crizotinib-responsive *ALK*-rearranged non-small cell lung cancer not detected by fluorescence *in situ* hybridization. Oncologist.

[27] Drilon A, Wang L, Arcila ME (2015). Broad, hybrid capture-based next-generation sequencing identifies actionable genomic alterations in lung adenocarcinomas otherwise negative for such alterations by other genomic testing approaches. Clin Cancer Res.

[28] Lim SM, Kim EY, Kim HR (2016). Genomic profiling of lung adenocarcinoma patients reveals therapeutic targets and confers clinical benefit when standard molecular testing is negative. Oncotarget.

[29] Liu LP, JL Liu, Shao D (2017). Comprehensive genomic profiling of lung cancer using a validated panel to explore therapeutic targets in East Asian patients. Cancer Sci.

[30] Ou S-Hl, Ali SM, Bogart J (2018). Characterization of 1, 233 NSCLCs with non-del19/L858R *EGFR* Mutations (EGFRm) Using Comprehensive Genomic Profiling (CGP). J Clin Oncol.

[31] Lin JJ, Zhu VW, Yoda S (2018). Impact of EML4-ALK variant on resistance mechanisms and clinical outcomes in ALK-positive lung cancer. J Clin Oncol.

[32] Song P, Zhang L, Shang CC (2018). Current status for anaplastic lymphoma kinase in non-small cell lung cancer. Chin J Lung Cancer.

[33] Gainor JF, Dardaei L, Yoda S (2016). Molecular mechanisms of resistance to first- and second-generation ALK inhibitors in *ALK*-rearranged lung cancer. Cancer Discov.

[34] Rossi G, Jocollé G, Conti A (2017). Detection of *ROS1* rearrangement in non-small cell lung cancer: current and future perspectives. Lung Cancer (Auckl).

[35] Zhu VW, Upadhyayb D, Schrock AB (2016). TPD52L1-ROS1, a new *ROS1* fusion variant in lung adenosquamous cell carcinoma identified by comprehensive genomic profiling. Lung Cancer.

[36] Ou S-HI, Chalmersb ZR, Azadaa MC (2015). Identification of a novel TMEM106B-ROS1 fusion variant in lung adenocarcinoma by comprehensive genomic profiling. Lung Cancer.

[37] Dagogo-Jack I, Martinez P, Yeap BY (2019). Impact of *BRAF* mutation class on disease characteristics and clinical outcomes in *BRAF*-mutant lung cancer. Clin Cancer Res.

[38] Canon J, Rex K, Saiki AY (2019). The clinical KRAS (G12C) inhibitor AMG 510 drives anti-tumour immunity. Nature.

[39] Hallin J, Engstrom LD, Hargis L (2020). The KRAS G12C inhibitor MRTX849 provides insight toward therapeutic susceptibility of *KRAS*-mutant cancers in mouse models and patients. Cancer Discov.

[40] Fakih M, O'Neil B, Price TJ (2019). Phase 1 study evaluating the safety, tolerability, pharmacokinetics (PK), and efficacy of AMG 510, a novel small molecule KRASG12C inhibitor, in advanced solid tumors. J Clin Oncol.

[41] Cocco E, Scaltriti M, Drilon A (2018). *NTRK* fusion-positive cancers and TRK inhibitor therapy. Nat Rev Clin Oncol.

[42] Demetri GD, Paz-Ares L, Farago AF (2018). LBA17-efficacy and safety of entrectinib in patients with *NTRK* fusion-positive tumors: pooled analysis of STARTRK-2, STARTRK-1 and ALKA-372-001. Ann Oncol.

[43] Drilon A, Laetsch TW, Kummar S (2018). Efficacy of larotrectinib in TRK fusion-positive cancers in adults and children. N Engl J Med.

[44] Schrock AB, Frampton GM, Suh J (2016). Characterization of 298 patients with lung cancer harboring MET exon 14 skipping alterations. J Thorac Oncol.

[45] Cancer Genome Atlas Research Network (2014). Comprehensive molecular profiling of lung adenocarcinoma. Nature.

[46] Drilon A, Cappuzzo F, Ou S-HI (2017). Targeting MET in lung cancer: will expectations finally be MET?, 12(1): 15-26. J Thorac Oncol.

[47] Camidge RD, Ou S-HI, Shapiro G (2014). Efficacy and safety of crizotinib in patients with advanced c-MET-amplified non-small cell lung cancer. J Clin Oncol.

[48] Drilon AE, Camidge DR, Ou S-HI (2016). Efficacy and safety of crizotinib in patients (pts) with advanced MET exon 14-altered non-small cell lung cancer (NSCLC). J Clin Oncol.

[49] Neal JW, Dahlberg SE, Wakelee HA (2015). Cabozantinib (C), Erlotinib (E) or the Combination (E+C) as second-or third-line therapy in patients with EGFR wild-type (wt) non-small cell lung cancer (NSCLC): A randomized phase 2 trial of the ECOG-ACRIN Cancer Research Group (E1512). J Clin Oncol.

[50] Scagliotti G, Pawel JV, Novello S (2015). Phase Ⅲ multinational, randomized, double-blind, placebo-controlled study of tivantinib (ARQ 197) plus erlotinib versus erlotinib alone in previously treated patients with locally advanced or metastatic nonsquamous non-small-cell lung cancer. J Clin Oncol.

[51] Yoshioka H, Azuma K, Yamamoto N (2015). A randomized, double-blind, placebo-controlled, phase Ⅲ trial of erlotinib with or without a c-Met inhibitor tivantinib (ARQ 197) in Asian patients with previously treated stage Ⅲb/IV nonsquamous nonsmall-cell lung cancer harboring wild-type epidermal growth factor receptor (ATTENTION Study). Ann Oncol.

[52] Chiba M, Togashi Y, Tomida S (2016). MEK inhibitors against MET-amplified non-small cell lung cancer. Int J Oncol.

[53] Lu S, Fang J, Cao LJ (2019). Preliminary efficacy and safety results of savolitinib treating patients with pulmonary sarcomatoid carcinoma (PSC) and other types of non-small cell lung cancer (NSCLC) harboring *MET* exon 14 skipping mutations. Cancer Res.

[54] Wolf J, Seto T, Han JY (2019). Capmatinib (INC280) in *MET* Dex14-mutated advanced non-small cell lung cancer (NSCLC): efficacy data from the phase Ⅱ GEOMETRY mono-1 study. J Clin Oncol.

[55] Paik PK, Veillon R, Cortot AB (2019). Phase Ⅱ study of tepotinib in NSCLC patients with *MET* ex14 mutations. J Clin Oncol.

[56] Stephens P, Hunter C, Bignell G (2004). Lung Cancer: Intragenic *ERBB2* kinase mutations in tumours. Nature.

[57] Arclia ME, Chaft JE, Nafa K (2012). Prevalence, clinicopathologic associations, and molecular spectrum of *ERBB2* (*HER2*) tyrosine kinase mutations in lung adenocarcinomas. Clin Cancer Res.

[58] Kris MG, Johnson BE, Berry LD (2014). Using multiplexed assays of oncogenic drivers in lung cancers to select targeted drugs. JAMA.

[59] Li BT, Shen R, Buonocore D (2018). Ado-trastuzumab emtansine in patients with *HER2* mutant lung cancers: results from a phase Ⅱ basket trial. J Clin Oncol.

[60] Smit EF, Nakagawa K, Nagasaka M (2020). Trastuzumab deruxtecan (T-DXd; DS-8201) in patients with *HER2*-mutated metastatic non-small cell lung cancer (NSCLC): Interim results of DESTINY-Lung01. J Clin Oncol.

[61] Gao G, Li X, Wang Q (2019). Single-arm, phase Ⅱ study of pyrotinib in advanced non-small cell lung cancer (NSCLC) patients with *HER2* exon 20 mutation. J Clin Oncol.

[62] Robichaux JP, Elamin YY, Tan Z (2018). Mechanisms and clinical activity of an EGFR and HER2 exon 20-selective kinase inhibitor in non-small cell lung cancer. Nat Med.

[63] Cappuzzo F, Bemis L, Varella-Garcia M (2006). *HER2* mutation and response to trastuzumab therapy in non-small-cell lung cancer. N Engl J Med.

[64] Greve JD, Teugels E, Geers C (2012). Clinical activity of afatinib (BIBW 2992) in patients with lung adenocarcinoma with mutations in the kinase domain of HER2/neu. Lung Cancer.

[65] Hyman DM, Piha-Paul SA, Rodon J (2017). Neratinib in *HER2* or *HER3* mutant solid tumors: SUMMIT, a global, multi-histology, open-label, phase 2 "basket" study. Cancer Res.

[66] Drilon A, Hu ZI, Lai G (2018). Targeting RET-driven cancers: lessons from evolving preclinical and clinical landscapes. Nat Rev Clin Oncol.

[67] Drilon A, Wang L, Hasanovic A (2013). Response to cabozantinib in patients with *RET* fusion-positive lung adenocarcinoma. Cancer Discov.

[68] Drilon A, Rekhtman N, Arcila M (2016). Cabozantinib in patients with advanced *RET*-rearranged non-small-cell lung cancer: an open-label, single-centre, phase 2, single-arm trial. Lancet Oncol.

[69] Lee SH, Lee JK, Ahn MJ (2017). Vandetanib in pretreated patients with advanced non-small cell lung cancer-harboring *RET* rearrangement: A phase Ⅱ clinical trial. Ann Oncol.

[70] Lin JJ, Kennedy E, Sequist LV (2016). Clinical activity of alectinib in advanced *RET*-rearranged non-small cell lung cancer. J Thorac Oncol.

[71] Hida T, Velcheti V, Reckamp KL (2019). A phase 2 study of lenvatinib in patients with *RET* fusion-positive lung adenocarcinoma. Lung Cancer.

[72] Li GG, Somwar R, Joseph J (2017). Antitumor activity of RXDX-105 in multiple cancer types with *RET* rearrangements or mutations. Clin Cancer Res.

[73] Gainor JF, Lee DH, Curigliano G (2019). Clinical activity and tolerability of BLU-667, a highly potent and selective RET inhibitor, in patients (pts) with advanced *RET*-fusion non-small cell lung cancer (NSCLC). J Clin Oncol.

[74] Drilon AE, Subbiah V, Oxnard GR (2018). A phase 1 study of LOXO-292, a potent and highly selective RET inhibitor, in patients with RET-altered cancers. J Clin Oncol.

[75] Gallant JN, Sheehan JH, Shaver TM (2015). EGFR kinase domain duplication (EGFR-KDD) is a novel oncogenic driver in lung cancer that is clinically responsive to afatinib. Cancer Disc.

[76] Zhu YC, Wang WX, Song ZB (2018). *MET-UBE2H* fusion as a novel mechanism of acquired EGFR resistance in lung adenocarcinoma. J Thorac Oncol.

[77] Konduri K, Gallant JN, Chae YK (2016). *EGFR* fusions as novel therapeutic targets in lung cancer. Cancer Discov.

[78] Lai J, Wang W, Xu C (2018). Real-world data in clinically non-small cell lung cancer (NSCLC) with Chinese population harboring *BRAF* gene fusions: a multicenter study. J Clin Oncol.

[79] Xu CW, Wang WX, Wang XJ (2019). Abstract 1597: real-world large-scale study kinase domain duplications across diverse tumor types in Chinese populations. Cancer Res.

[80] Ross JS, Wang K, Chmielecki J (2016). The distribution of *BRAF* gene fusions in solid tumors and response to targeted therapy. Int J Cancer.

[81] Zhu YC, Wang WX, Xu CW (2019). A patient with lung adenocarcinoma with *BRAF* gene fusion and response to vemurafenib. Clin Lung Cancer.

[82] Wang WX, Xu C, Chen Y (2018). *MET* gene fusions in non-small cell lung cancer (NSCLC) in the Chinese population: a multicenter study. J Clin Oncol.

[83] Davies KD, Ng TL, Estrada-Bernal A (2017). Dramatic response to crizotinib in a patient with lung cancer positive for an *HLA-DRB1-MET* gene fusion. JCO Precis Oncol.

[84] Cho JH, Ku BM, Sun JM (2018). *KIF5B-MET* gene rearrangement with robust antitumor activity in response to crizotinib in lung adenocarcinoma. J Thorac Oncol.

[85] Xu CW, Wang WX, Zhang QX (2019). Real-world large-scale study of *ERBB2* gene fusions and its response to afatinib in Chinese non-small cell lung cancer (NSCLC): a multicenter study. J Clin Oncol.

[86] Garon EB, Rizvi NA, Hui R (2015). Pembrolizumab for the treatment of non-small-cell lung cancer. N Engl J Med.

[87] Remon J, Passiglia F, Ahn MJ (2020). Immune checkpoint inhibitors in thoracic malignancies: review of the existing evidence by an IASLC expert panel and recommendations. J Thorac Oncol.

[b88] 88US Food and Drug Administration. FDA approves first cancer treatment for any solid tumor with a specific genetic feature. FDA News Release (2017). Available from: https://www.fda.gov/NewsEvents/Newsroom/PressAnnouncements/ucm560167.htm. Accessed August 31, 2020.

[89] Rizvi NA, Cho BC, Reinmuth N (2019). Blood tumor mutational burden (bTMB) and tumor PD-L1 as predictive biomarkers of survival in MYSTIC: first-line durvalumab (D) 6 tremelimumab (T) versus chemotherapy (CT) in metastatic (m) NSCLC. J Clin Oncol.

[90] Singal G, Miller PG, Agarwala V (2019). Association of patient characteristics and tumor genomics with clinical outcomes among patients with non-small cell lung cancer using a clinicogenomic database. JAMA.

[91] Castellanos E, Snider J, Ali SM (2019). Tumor mutational burden (TMB) and PD-L1 expression as predictors of response to immunotherapy (IO) in NSCLC. J Clin Oncol.

[92] Carbone DP, Reck M, Paz-Ares L (2017). First-line nivolumab in stage IV or recurrent non-small-cell lung cancer. N Engl J Med.

[93] Hellmann MD, Ciuleanu TE, Pluzanski A (2018). Nivolumab plus ipilimumab in lung cancer with a high tumor mutational burden. N Engl J Med.

[b94] 94US Food and Drug Administration. FDA grants pembrolizumab priority review for TMB-high tumors. FDA News Release (2020). Available from: https://www.targetedonc.com/view/fda-grants-pembrolizumab-priority-review-for-tmbhigh-tumors. Accessed August 31, 2020.

[95] Gandara DR, Pawel JV, Mazieres J (2018). Atezolizumab treatment beyond progression in advanced NSCLC: results from the randomized, phase Ⅲ OAK study. J Thorac Oncol.

[96] Socinski M, Velcheti V, Mekhail T (2019). LBA83-Final efficacy results from B-F1RST, a prospective phase Ⅱ trial evaluating blood-based tumour mutational burden (bTMB) as a predictive biomarker for atezolizumab (atezo) in 1L non-small cell lung cancer (NSCLC). Ann Oncol.

[97] Dong ZY, Zhong WZ, Zhang XC (2017). Potential predictive value of *TP53* and *KRAS* mutation status for response to PD-1 blockade immunotherapy in lung adenocarcinoma. Clin Cancer Res.

[98] Ross JS, Goldberg ME, Albacker LA (2017). Immune checkpoint inhibitor (ICPI) efficacy and resistance detected by comprehensive genomic profiling (CGP) in non-small cell lung cancer (NSCLC). Ann Oncol.

[99] Chen J, Jiang D, Huang F (2019). Advances of the correlation between driver gene status and immunotherapy in non-small cell lung cancer. Zhongguo Fei Ai Za Zhi.

[100] Ferrara R, Mezquita L, Texier M (2018). Hyperprogressive disease in patients with advanced non-small cell lung cancer treated with PD-1/PD-L1 inhibitors or with single-agent chemotherapy. JAMA Oncol.

[101] Saâda-Bouzid E, Defaucheux C, Karabajakian A (2017). Hyperprogression during anti-PD-1/PD-L1 therapy in patients with recurrent and/or metastatic head and neck squamous cell carcinoma. Ann Oncol.

[102] Singavi AK, Menon S, Kilari D (2017). Predictive biomarkers for hyper-progression (HP) in response to immune checkpoint inhibitors (ICI)-analysis of somatic alterations (SAs). Ann Oncol.

[103] Adams DR, Eng CM (2018). Next-generation sequencing to diagnose suspected genetic disorders. N Engl J Med.

[104] Gagan J, Van Allen EM (2015). Next-generation sequencing to guide cancer therapy. Genome Med.

[105] Dong L, Wang W, Li A (2015). Clinical next generation sequencing for precision medicine in cancer. Curr Genomics.

[b106] 106National Medical Products Administration.国家药品监督管理局. Available from: http://app1.nmpa.gov.cn. Accessed August 31, 2020.

[b107] 107US Food and Drug Administration. FDA announces approval, CMS proposes coverage of first breakthrough-designated test to detect extensive number of cancer biomarkers[EB/OL]. Available from: https://www.fda.gov/NewsEvents/Newsroom/PressAnnouncements/ucm587273.htm. Accessed August 31, 2020.

[108] Frampton GM, Fichtenholtz A, Otto GA (2013). Development and validation of a clinical cancer genomic profiling test based on massively parallel DNA sequencing. Nat Biotechnol.

[109] Swisher EM, Lin KK, Oza AM (2017). Rucaparib in relapsed, platinum-sensitive high-grade ovarian carcinoma (ARIEL2 Part 1): an international, multicentre, open-label, phase 2 trial. Lancet Oncol.

[110] Ready N, Hellmann MD, Awad MM (2019). First-line nivolumab plus ipilimumab in advanced non-small-cell lung cancer (CheckMate 568): outcomes by programmed death ligand 1 and tumor mutational burden as biomarkers. J Clin Oncol.

[111] Gargis AS, Kalman L, Berry MW (2012). Assuring the quality of next-generation sequencing in clinical laboratory practice. Nat Biotechnol.

[b112] 112Center for Medical Device Evaluation. NMPA, General technical guidelines for registration review of tumor related mutation gene detection reagents (high-throughput sequencing) performance evaluation.国家药品监督管理局医疗器械技术审评中心, 肿瘤相关突变基因检测试剂(高通量测序法)性能评价通用技术注册审查指导原则[2019年第83号]. Available from: https://www.nmpa.gov.cn/ylqx/ylqxggtg/ylqxzhdyz/20191205093801407.html?type=pc&m=. Accessed August 31, 2020.

[113] Zhang X, Liang Z, Wang S (2019). Application of next-generation sequencing technology to precision medicine in cancer: joint consensus of the tumor biomarker committee of the Chinese Society of Clinical Oncology. Cancer Biol Med.

[114] Francis G, Stein S (2015). Circulating cell-free tumour DNA in the management of cancer. Int J Mol Sci.

[115] Remon J, Caramella C, Jovelet C (2017). Osimertinib benefit in *EGFR*-mutant NSCLC patients with T790M-mutation detected by circulating tumour DNA. Ann Oncol.

[116] Perakis S, Speicher MR (2017). Emerging concepts in liquid biopsies. BMC Med.

[117] Siravegna G, Marsoni S, Siena S (2017). Integrating liquid biopsies into the management of cancer. Nat Rev Clin Oncol.

[118] Rolfo C, Mack PC, Scagliotti GV (2018). Liquid biopsy for advanced non-small cell lung cancer (NSCLC): a statement paper from the IASLC. J Thorac Oncol.

[119] Merker JD, Oxnard GR, Compton C (2018). Circulating tumor DNA analysis in patients with cancer: American Society of Clinical Oncology and College of American Pathologists Joint Review. Arch Pathol Lab Med.

[120] Heitzer E, Haque IS, Roberts CE (2019). Current and future perspectives of liquid biopsies in genomics-driven oncology. Nat Rev Genet.

[121] Song Y, Hu C, Xie Z (2020). Circulating tumor DNA clearance predicts prognosis across treatment regimen in a large real-world longitudinally monitored advanced non-small cell lung cancer cohort. Transl Lung Cancer Res.

[122] Wang Z, Cheng Y, An T (2018). Detection of *EGFR* mutations in plasma circulating tumour DNA as a selection criterion for first-line gefitinib treatment in patients with advanced lung adenocarcinoma (BENEFIT): A phase 2, single-arm, multicentre clinical trial. Lancet Respir Med.

[123] Jovelet C, Ileana E, Le Deley MC (2016). Circulating cell-free tumor DNA analysis of 50 genes by next-generation sequencing in the prospective MOSCATO trial. Clin Cancer Res.

[124] Aldea M, Hendriks L, Mezquita L (2020). Circulating tumor DNA analysis for patients with oncogene-addicted NSCLC with isolated central nervous system progression. J Thorac Oncol.

[125] Li YS, Jiang BY, Yang JJ (2018). Unique genetic profiles from cerebrospinal fluid cell-free DNA in leptomeningeal metastases of *EGFR*-mutant non-small-cell lung cancer: a new medium of liquid biopsy. Ann Oncol.

[126] Ying S, Ke H, Ding Y (2019). Unique genomic profiles obtained from cerebrospinal fluid cell-free DNA of non-small cell lung cancer patients with leptomeningeal metastases. Cancer Biol Ther.

[127] Meichen Li, Delan Li, Xue Hou (2020). Utilizing phenotypic characteristics of metastatic brain tumors to predict the probability of circulating tumor DNA detection from cerebrospinal fluid. J Clin Oncol.

[128] Nevel KS, Wilcox JA, Robell LJ (2018). The utility of liquid biopsy in central nervous system malignancies. Curr Oncol Rep.

[129] Mouliere F, Mair R, Chandrananda D (2018). Detection of cell-free DNA fragmentation and copy number alterations in cerebrospinal fluid from glioma patients. EMBO Mol Med.

[130] Liu L, Shao D, Deng Q (2018). Next generation sequencing-based molecular profiling of lung adenocarcinoma using pleural effusion specimens. J Thorac Dis.

[131] Guo Z, Xie Z, Shi H (2019). Malignant pleural effusion supernatant is an alternative liquid biopsy specimen for comprehensive mutational profiling. Thoracic Cancer.

[132] Villatoro S, Mayo-de-Las-Casas C, Jordana-Ariza N (2019). Prospective detection of mutations in cerebrospinal fluid, pleural effusion, and ascites of advanced cancer patients to guide treatment decisions. Mol Oncol.

[133] Bettegowda C, Sausen M, Leary RJ (2014). Detection of circulating tumor DNA in early- and late-stage human malignancies. Sci Transl Med.

[134] Jahangiri L, Hurst T (2019). Assessing the concordance of genomic alterations between circulating-free DNA and tumour tissue in cancer patients. Cancers (Basel).

[135] Klempner SJ, Bazhenova LA, Braiteh FS (2015). Emergence of *RET* rearrangement coexisting with activated *EGFR* mutation in *EGFR*-mutated NSCLC patients who had progressed on first- or second-generation EGFR TKI. Lung Cancer.

[136] Ou S-HI, Horn L, Cruz M (2017). Emergence of *FGFR3-TACC3* fusions as a potential by-pass resistance mechanism to EGFR tyrosine kinase inhibitors in *EGFR* mutated NSCLC patients. Lung Cancer.

[137] Yu HA, Suzawa K, Jordan E (2018). Concurrent alterations in *EGFR*-mutant lung cancers associated with resistance to EGFR kinase inhibitors and characterization of MTOR as a mediator of resistance. Cancer Res.

[138] Shaw AT, Martini JF, Besse B (2018). Efficacy of lorlatinib in patients (pts) with advanced ALK-positive non-small cell lung cancer (NSCLC) and ALK kinase domain mutations. Cancer Res.

[139] Roys A, Chang X, Liu Y (2019). Resistance mechanisms and potent-targeted therapies of ROS1-positive lung cancer. Cancer Chemother Pharmacol.

[140] Dziadziuszko R, Le AT, Wrona A (2016). An activating *KIT* mutation induces crizotinib resistance in ROS1-positive lung cancer. J Thorac Oncol.

[141] Chen YF, Hsieh MS, Wu SG (2016). Efficacy of pemetrexed-based chemotherapy in patients with *ROS1* fusion-positive lung adenocarcinoma compared with in patients harboring other driver mutations in East Asian populations. J Thorac Oncol.

[142] Facchinetti F, Loriot Y, Kuo MS (2016). Crizotinib-resistant *ROS1* mutations reveal a predictive kinase inhibitor sensitivity model for *ROS1*- and *ALK*-rearranged lung cancers. Clin Cancer Res.

[143] Rudin CM, Hong K, Streit M (2013). Molecular characterization of acquired resistance to the BRAF inhibitor dabrafenib in a patient with *BRAF*-mutant non-small-cell lung cancer. J Thorac Oncol.

[144] Niemantsverdriet M, Schuuring E, Elst AT (2018). *KRAS* mutation as a resistance mechanism to BRAF/MEK inhibition in NSCLC. J Thorac Oncol.

[145] Abravanel DL, Nishino M, Sholl LM (2018). An acquired *NRAS* Q61K mutation in *BRAF* V600E-mutant lung adenocarcinoma resistant to dabrafenib plus trametinib. J Thorac Oncol.

[146] Facchinetti F, Lacroix L, Mezquita L (2020). Molecular mechanisms of resistance to BRAF and MEK inhibitors in BRAF (V600E) non-small cell lung cancer. Eur J Cancer.

[147] Drilon A, Nagasubramanian R, Blake JF (2017). A next-generation TRK kinase inhibitor overcomes acquired resistance to prior TRK kinase inhibition in patients with TRK fusion-positive solid tumors. Cancer Discov.

[148] Zhai D, Deng W, Huang J (2017). TPX-0005, an ALK/ROS1/TRK inhibitor, overcomes multiple resistance mechanisms by targeting SRC/FAK signaling. Cancer Res.

[149] Fuse MJ, Okada K, Oh-Hara T (2017). Mechanisms of resistance to NTRK inhibitors and therapeutic strategies in *NTRK1*-rearranged cancers. Mol Cancer Ther.

[150] Doebele RC, Dziadziusko R, Drilon A (2019). LBA28-genomic landscape of entrectinib resistance from ctDNA analysis in STARTRK-2. Ann Oncol.

[151] Ou SI, Young L, Schrock AB (2017). Emergence of preexisting *MET* Y1230C mutation as a resistance mechanism to crizotinib in NSCLC with MET exon 14 skipping. J Thorac Oncol.

[152] Heist RS, Sequist LV, Borger D (2016). Acquired resistance to crizotinib in NSCLC with MET exon 14 skipping. J Thorac Oncol.

[153] Guo R, Offin M, Brannon AR (2019). MET inhibitor resistance in patients with MET exon 14-altered lung cancers. J Clin Oncol.

[154] Ding G, Wang J, Ding P (2019). Case report: HER2 amplification as a resistance mechanism to crizotinib in NSCLC with MET exon 14 skipping. Cancer Biol Ther.

[155] Koga T, Kobayashi Y, Tomizawa K (2018). Activity of a novel HER2 inhibitor, poziotinib, for *HER2* exon 20 mutations in lung cancer and mechanism of acquired resistance: an *in vitro* study. Lung Cancer.

[156] Chuang JC, Stehr H, Liang Y (2017). *ERBB2*-mutated metastatic non-small cell lung cancer: response and resistance to targeted therapies. J Thorac Oncol.

[157] Liu X, Shen T, Mooers BHM (2018). Drug resistance profiles of mutations in the RET kinase domain. Br J Pharmacol.

[158] Nakaoku T, Kohno T, Araki M (2018). A secondary *RET* mutation in the activation loop conferring resistance to vandetanib. Nat Commun.

[159] Dagogo-Jack I, Stevens SE, Lin JJ (2018). Emergence of a *RET* V804M gatekeeper mutation during treatment with vandetanib in *RET*-rearranged NSCLC. J Thorac Oncol.

[160] Ramalingam SS, Cheng Y, Zhou C (2018). Mechanisms of acquired resistance to first-line osimertinib: preliminary data from the phase Ⅲ FLAURA study. Ann Oncol.

[161] Guo J, Guo L, Sun L (2018). Capture-based ultra-deep sequencing in plasma ctDNA reveals the resistance mechanism of ALK inhibitors in a patient with advanced ALK-positive NSCLC. Cancer Biol Ther.

[162] Jin W, Shan B, Liu H (2019). Acquired mechanism of crizotinib resistance in NSCLC with MET exon 14 skipping. J Thorac Oncol.

[163] Bahcall M, Sim T, Paweletz CP (2016). Acquired *MET*D1228V mutation and resistance to MET inhibition in lung cancer. Cancer Discov.

